# Interfacial Adsorption Interactions of Dyes and Chitosan/Activated Carbon@Curcumin Derivatives in Single-Component and Binary Solutions

**DOI:** 10.1021/acs.langmuir.4c04769

**Published:** 2025-01-27

**Authors:** Angeliki A. Kouzoutzoglou-Efremidou, Athanasia K. Tolkou, Konstantinos N. Maroulas, Ramonna I. Kosheleva, Ioannis A. Katsoyiannis, George Z. Kyzas

**Affiliations:** † Hephaestus Laboratory, School of Chemistry, Faculty of Sciences, Democritus University of Thrace, GR-65404 Kavala, Greece; ‡ Laboratory of Chemical and Environmental Technology, Department of Chemistry, Aristotle University of Thessaloniki, GR-54124 Thessaloniki, Greece

## Abstract

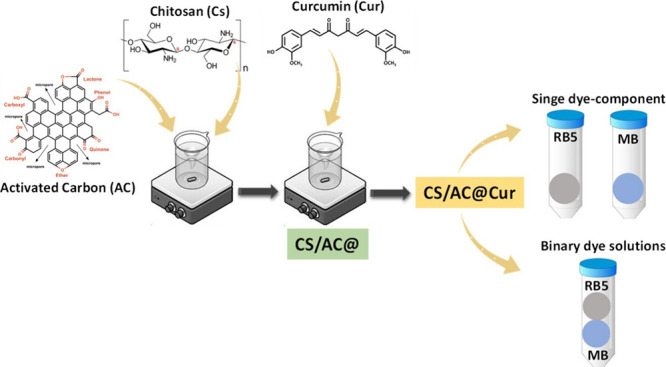

The remediation of wastewaters contaminated with dyes (discharged mainly from industry) is very important for preserving environmental quality and human health. In this study, a new composite chitosan (CS)-based adsorbent combined with activated carbon (AC) and curcumin (Cur) (abbreviated hereafter as CS/AC@Cur) in three different ratios (12.5%, 25%, and 50%) was synthesized for the removal of anionic [reactive black 5 (RB5)] and cationic [methylene blue (MB)] dyes in single-component or binary systems. The synthesized materials were completely characterized through Fourier transform infrared spectroscopy, scanning electron microscopy, Brunauer–Emmett–Teller analysis, and X-ray diffraction. Specifically, a decrease in the surface area of CS/AC@ was observed with the addition of curcumin, from 163 to 18 m^2^/g, for all CS/AC@Cur derivatives. In terms of the adsorption results, the optimal derivative for the removal of both RB5 and MB was found to be CS/AC@Cur50%, providing 93% removal at pH 2.0 ± 0.1 for RB5 and 54% removal at the optimum pH of 9.0 ± 0.1 due to electrostatic attractions. The Elovich and pseudo-second-order kinetic model, with a correlation coefficient *R*^2^ of >0.98, better tailored the results, indicating that adsorption was controlled by chemisorption. In addition, the Sips (Langmuir–Freundlich) isotherm model fitted better to the results, with calculated capacities of 338 and 307 mg/g for RB5 and MB, respectively. The thermodynamic analysis showed a spontaneous and endothermic procedure, with chemisorption as the main mechanism. Reuse experiments showed that the removal efficiency was retained at high levels, while stability studies revealed that the adsorbent retains its structural integrity, even at extreme pH values. Finally, the adsorption of RB5 and MB in a mixed solution was investigated, providing a competitive effect between the anionic and cationic dyes.

## Introduction

Wastewaters containing dyes and pigments, often originating from textile, pulp and paper, plastic, and food industries, produce various pollutants.^1^ Dyes have serious environmental harmful effects, as because of their chemical construction, they are thermally stable and photostable, while they are also toxic in nature and resistant to biodegradation.^2^ As a result, in addition to remaining in the environment for a long time, they have the ability due to the absorption and reflection of incident radiation from the sun to penetrate the water and in this way affect the process of photosynthesis of algae and consequently the food chain. Many dyes have been found to cause cancer and teratogenesis, as they are mutagenic and toxic to living creatures, and to exhibit aromaticity.^2^ Hence, eliminating dyes from wastewater is essential, as it is one of the most important industrial wastes worldwide, given that ∼1 billion tons of dye wastewater is disposed of annually. In countries such as China, Bangladesh, Malaysia, and Indonesia, which still dominate the dye industry, this problem is more evident.^3^

The removal of dyes from wastewater is based on chemical, physical, and biological techniques,^1^ including ozonation,^4^ chemical oxidation,^5^ coagulation–flocculation,^6^ adsorption,^7^ and membrane separation.^8^ Among them, adsorption is a low-cost technique and one of the commonly used. In addition, adsorption has advantages over other techniques due to its ease of application and design, as well as ease of recovery, providing a high pollutant removal efficiency.^9^ Furthermore, one of the undeniable advantages of adsorption is the possibility of increasing the adsorption properties through several surface modifications or improving conditions such as the temperature, pH, and contact time.

There is a wide array of adsorbents that are used for dye removal, such as hydroxide nanosheets,^10^ metal–organic frameworks (MOFs),^11,12^ zeolites,^13^ chitosan,^7^ magnetic nanocomposites,^14^ graphene oxide,^15^ fruit peels,^16^ inorganic–organic hybrid nanocomposites,^17^ activated carbon,^18^ etc. However, in practice, there are many limitations to the use of these adsorbents, such as the high cost of some of them, the low adsorption capacity, difficulty in regeneration, long contact times, etc.^10^ Among these, activated carbon continues to be one of the highly effective adsorbents, particularly in the removal of organic pollutants, i.e., dyes, thanks to its unique assets, such as its porous form, available functional groups, and ability to be reused.^19^ Moreover, chitosan, which resulted from the deacetylation of chitin, is an abundant aminopolysaccharide (biopolymer) that recently was identified as one of the most studied adsorptive materials.^20^ Its interaction with organic molecules such as dyes^7^ is facilitated by amino and hydroxy groups that exist in chitosan’s chemical structure. In addition, chitosan is biodegradable, nontoxic, and readily available.^21^ Blachino et al. studied the application of chitosan–silica hybrid composites for the removal of azo dyes.^22^ Nevertheless, several advanatges, such as its low porosity, small surface area, high crystallinity, and hydrolyzability in acidic medium applications,^23^ make its modification necessary. Furthermore, curcumin, the main curcuminoid in turmeric, which is used in Indian curries, is a polyphenol and is considered to be a natural adsorbent, which has been widely examined for water treatment.^24^ On the contrary, several drawbacks, such as solubility and lack of stability leading to degradation in heat and sunlight, led researchers to graft modification and encapsulation of curcumin.^25^ In addition, several curcumin-based adsorbents were studied with respect to the removal of dyes.^26^

Among the wide range of dyes found in wastewater, two types of dyes are selected as pollutants in this study. The first one is reactive black 5 (RB5), which is an anionic azo dye, widely used in the coloring of cellulose-based fiber.^27^ These dyes form a strong chemical bond when in contact with fibers, creating a covalent bond. Nevertheless, the presence of this class of dyes in industrial processes endangers aquatic ecosystems. The other dye is methylene blue (MB), which is a basic dye, widely used in industries such as dyeing, printing, and pharmaceuticals.^28^ Cationic dyes form strong bonds due to their attraction to the negatively charged surfaces of materials.^29^ However, it has been found to affect the central nervous system, causing skin diseases. Subsequently, the presence of MB in water poses a risk to both aquatic ecosystems and humans. Hence, CS is used as an adsorbent, due to possible electrostatic attraction that may occur between the −SO_3_^–^ and −NH_2_ groups of RB5 and the protonated −NH_3_^+^ groups on chitosan, thus enhancing adsorption. In addition, nitrogen (N), sulfur (S), and oxygen (O) ions from both RB5 and MB can form hydrogen bonds with chitosan, facilitating chelation.^21^ In addition, possible π–π interaction between curcumin and anionic RB5 would promote adsorption.^30^ Moreover, curcumin is often used as an anti-infective, immunomodulatory, and anti-inflammatory substance, possibly also giving the material disinfectant capabilities. Therefore, the combination of CS, AC, and Cur in a unique material for wastewater treatment is proposed, taking advantage of the benefits of each substitute.

Therefore, this research focuses on the synthesis of effective chitosan-based adsorbents, combined initially with activated carbon and then treated with curcumin, denoted hereafter as CS/AC@Cur derivatives, for the removal of anionic and cationic dyes in single-component or binary systems. This modification targets the distinct strengths and properties of interfacial adsorption interactions between the adsorbent (dye molecules) and the adsorbate (chitosan-based composites). According to a recent literature review, the use of composite adsorbents of chitosan, activated carbon, and curcumin in a single reagent for the removal of RB5 and MB has not been examined. The innovation of this work is the development of a viable and economical approach for the synthesis of a composite adsorbent for the eventual effective treatment of wastewaters containing dyes. In accordance with the established practice, to evaluate the adsorption process, the effects of the initial pH of the solution, the dosage of the adsorbent, the contact time, the initial concentration of dyes, and the temperature were studied. For a better understanding and interpretation of the adsorption process, kinetic and isothermal models were applied as well as thermodynamic analysis. Finally, the materials were fully characterized before and after their application.

## Materials and Methods

### Materials

As model dyes, RB5 (purity of ≥50%), an anionic dye from Kahafix, and MB (purity of ≥95%), a cationic dye purchased from Sigma-Aldrich-Merck KGaA (Darmstadt, Germany), were used. For the synthesis of the adsorbents, chitosan (CS) (310–375 kDa, DDA > 75%), glutaraldehyde (GLA) (50 wt % in H_2_O), and activated carbon (AC) were supplied by Sigma-Aldrich-Merck KGaA. Additionally, acetic acid (≥99%) purchased from Fisher Chemicals (Hampton, NH) was used, as well as sodium tripolyphosphate (TPP, Na_5_O_10_P_3_, molar mass of 367 g/mol) from Alfa Aesar, Thermo Fisher Scientific Inc., as a cross-linker. The last additive, curcumin (Cur) (>70%), was purchased from Glentham Life Sciences. To adjust the pH, even diluted solutions of 37% HCl from Panreac, AppliChem (Barcelona, Spain), or NaOH (≥97.0% ACS NaOH pellets) from Sigma-Aldrich-Merck KGaA were used. In addition, NaCl and NaNO_3_ supplied by Sigma-Aldrich-Merck KGaA were also used to examine the effect of salt concentration in dye removal.

### Synthesis of CS/AC@Cur Derivatives

CS/AC@Cur derivatives were synthesized following the suggested procedure described below. First, an appropriate amount of AC was added to a 1% (w/v) chitosan solution, prepared on the basis of a previous study of ours,^31^ to achieve different molar ratios, and the mixture was stirred at 50 °C. After preliminary experiments and previously published work,^31^ to determine the optimum ratio for AC addition, a 2:1 CS:AC ratio (1:1 and 2:1 ratios were also examined) was found to be optimal and chosen for the synthesis of the materials. A Cur solution was then prepared and added to the CS/AC@ solution described above, under vigorous stirring, with different contents using acetone as the solvent, followed by heating at 50 °C, until the acetone evaporated. The selection of Cur ratio (12.5%, 25%, and 50% by weight relative to chitosan) was based on the literature and preliminary experiments that are not described here, concluding in the three best ratios. Karthikeyan et al. tested different CS:Cur ratios for antibacterial properties, selecting 25% as the best.^32^ Rezagholizade-shirvan et al.^33^ employed multiple Cur:Cs ratios and found that CS nanoparticles can be loaded with ∼50 wt % Cur to successfully remove Cr(VI) from wastewaters. In another study by Jebarani et al.,^34^ membranes were employed with a curcumin:multiwalled carbon nanotube ratio of 10% for the removal of Congo red dye. Then, after the GLA solution was added, colloid solution gels were obtained, which were stored for 24 h in a freezer. They were then lyophilized at −104 °C for 48 h to form the aerogels. These aerogels were ground into fine powder and purified in a Soxhlet apparatus (using a water/methanol mixture for 24 h). Thus, after this synthesis, the new composite derivatives emerged, henceforth termed CS/AC@Cur12.5%, CS/AC@Cur25%, and CS/AC@Cur50%, respectively.

### Analytical Techniques

After the completion of batch adsorption experiments, a sample is drawn through a syringe from the supernatant of each Falcon tube, which is then filtered through a nylon membrane filter (0.45 μm). The determination of both the initial and the final concentration of each dye is achieved by the corresponding absorbance and matching it to the reference curve obtained by a UV–vis spectrophotometer (WTW Spectroflex 6100, Weilheim, Germany) at λ_max_ values of 603 and 664 nm for RB5 and MB, respectively.^35^

### Characterization Techniques

To analyze the surface morphology and to determine the surface chemical bonds and functional groups of the synthesized CS/AC@Cur derivatives, several techniques were applied. Among them, porosimetry with Brunauer–Emmett–Teller (BET) analysis software (Quantachrome NovaWin - Data Acquisition and Reduction for NOVA instruments ©1994–2012, Quantachrome Instruments, version 11.02), scanning electron microscopy (SEM) (Jeol JSM-6390 LV), Fourier transform infrared spectroscopy (FTIR) (PerkinElmer, New York, NY), and X-ray diffraction (XRD) (Rigaku MiniFlex II, Rigaku Corp., Tokyo, Japan) assessed the crystallinity using a Bruker D8 FOCUS diffractometer.

### Batch Adsorption Experiments

To evaluate the adsorption of dyes from aqueous solutions by CS/AC@Cur derivatives, RB5 and MB dye solutions of various concentrations were made by diluting a 1000 mg/L stock dye solution with distilled water. The adsorption study was performed under various conditions, including different solution pH values (2.0, 5.0, 7.0, and 9.0), initial dye concentrations (20–500 mg/L), temperatures (293–323 K), contact times (0–240 min and 24 h for equilibrium), and adsorbent amounts (0.2–1.0 g/L), in 10 mL of a dye solution with 80 rpm agitation. It should be noted that most of the parameters were selected according to the literature.^9^ Specifically, dye concentrations in textile wastewaters are reported to cover a wide range, depending on the type of industry producing the waste, presenting an average of 10–250 mg/L and in some individual cases higher than this range.^36^

The percentage removal [*R* (percent)] of RB5 and MB was determined with eq 1, while the adsorption capacity of the adsorbent [*Q*_e_ (milligrams per gram)] was calculated with eq 2:
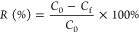
1
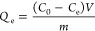
2where *C*_0_ is the initial RB5 and MB concentration in milligrams per liter, *C*_f_ is the final RB5 and MB concentration in milligrams per liter, *C*_e_ is the equilibrium RB5 and MB concentration in milligrams per liter, *V* is the solution’s volume in liters, and *m* is the adsorbent’s mass in grams.

### Adsorption Isotherms

Adsorption isotherms are used to examine the interface between the adsorbent and the adsorbent surface. Many isotherm models are used widely in liquid–solid adsorption systems, and among them, the most common and best fitted to the isotherm data are the Langmuir^37^ and Freundlich^38^ isotherm models. The Langmuir isotherm assumes monolayer adsorption and assumes that there are no interactions between the active sites and the adsorbates, i.e., dyes in this study, indicating probably that the amount of dyes absorbed does not affect the degree of uptake. Furthermore, the Freundlich isotherm considers multilayer adsorption and considers that the available active sites are energetically heterogeneous.^38^ In addition, the Langmuir–Freundlich (L–F) (or Sips) isotherm model^39^ was also applied to fit the data of this study. The combination of the Langmuir and Freundlich models is described by the Sips isotherm, which is a three-parameter model.

Equations 3–5 are used to fit and calculate the relative parameters for the selected isotherm models:
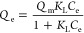
3
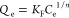
4
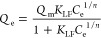
5where *Q*_e_ is the adsorption capacity of the adsorbent in milligrams per gram, *Q*_m_ is the maximum adsorption capacity in milligrams per gram, *K*_L_ is the constant for energy associated with the adsorption of RB5 and MB in liters per milligram, *K*_F_ is the constant that pertains to the adsorption capacity in (mg/g)·(L/mg)^1/*n*^, *K*_LF_ is the constant for heterogeneous surfaces in liters per gram, and 1/*n* is the constant linked to the adsorption intensity or a surface’s heterogeneity (0 < *n* < 1).

### Kinetics Experiments

Kinetics provides additional information for predicting the potential adsorption mechanism. Therefore, to analyze the performance of this phenomenon, a number of models such as pseudo-first-order (PFO), or as it is also called Lagergren, and pseudo-second-order (PSO) kinetic models are mentioned.^40^ In addition, Elovich^34^ and intraparticle diffusion (IPD)^41^ kinetic models were also examined to improve our understanding of the adsorption mechanism. The relative parameters for the selected kinetic models are calculated by eq 6 for the PFO model, eq 7 for the PSO model, eq 8 for IPD, and eq 9 for the Elovich model to better comprehend the adsorption of RB5 and MB.
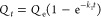
6
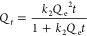
7
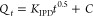
8
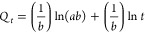
9where *Q*_e_ is the adsorption capacity of the adsorbent in milligrams per gram, *Q_t_* is the adsorption capacity of the adsorbent in milligrams per gram at time *t*, *k*_1_ is the rate constant for the PFO model in inverse minutes, *k*_2_ is the rate constant for the PSO model in grams per milligram per minute, *t* is the the contact time in minutes, *K*_IPD_ is the rate constant in milligrams per gram per half-minute, *C* gives an idea about the thickness of the boundary layer in milligrams per gram, *a* is the initial adsorption rate in milligrams per gram per second, *b* is the Elovich constant regarding the extent of surface coverage and activation energy for chemisorption (grams per milligram), and *t* is the the contact time in minutes.

### Thermodynamics

Thermodynamic parameters, such as the change in the Gibbs free energy (Δ*G*°, kilojoules per mole), enthalpy (Δ*H*°, kilojoules per mole), and entropy (Δ*S*°, kilojoules per mole per kelvin), are measured to primarily assess the nature of the adsorption process. Consequently, the experiments were conducted at 298, 308, 318, and 338 K, and eqs 10–13 were applied:
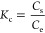
10
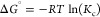
11
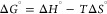
12
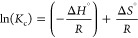
13where *K*_c_ is the thermodynamic constant, *R* is the universal gas constant (8.314 J mol^–1^ K^–1^), and *C*_s_ is the amount adsorbed on the solid at equilibrium in milligrams per liter.

## Results and Discussion

### Effect of the Initial Solution pH and Comparison of Materials

**Figure 1:**
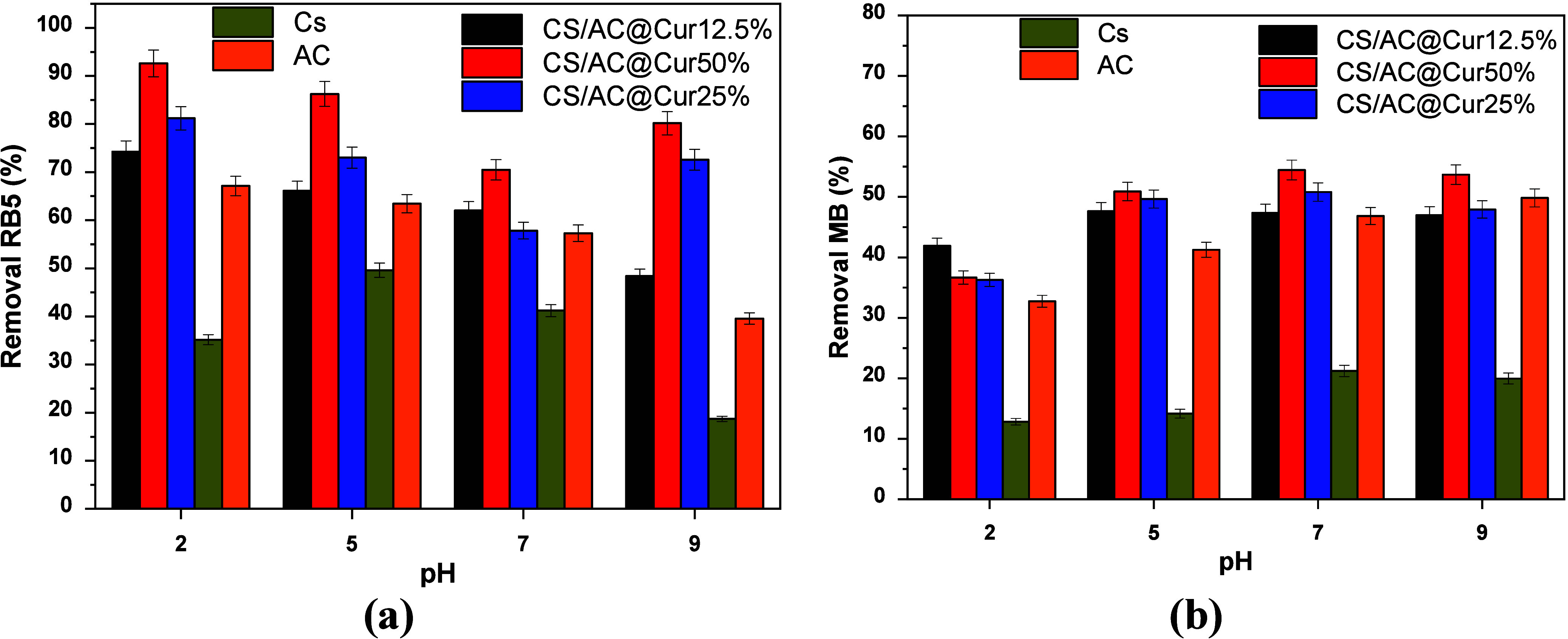
Effect of pH (initial) and comparison of materials for (a) RB5 and (b) MB adsorption. Experimental conditions: initial concentration of dyes, 100 mg/L; dose of adsorbents, 0.5 g/L; working temperature, 293 K; and duration of the experiment, 24 h.

In the treatment of water and wastewater, the primary factor to be studied is the pH of the initial solution, as it can affect the performance of the adsorbents. For this reason, a wide pH range (2.0 to 9.0 ± 0.1) was examined. A constant initial dye concentration (100 mg/L) and a constant dosage of adsorbents (0.5 g/L) were applied. The three different ratios of CS/AC@Cur derivatives, i.e., 12.5%, 25%, and 50%, were used, and these derivatives were compared as adsorbents for the removal of both RB5 (Figure 1a) and MB (Figure 1b). GLA cross-linked CS and pristine AC were selected as the control specimens. The application of these curcumin:chitosan ratios in the synthesis of the materials was initially chosen according to the limited existing literature, in which Karthikeyan et al. synthesized a hybrid nanomaterial used for antibacterial treatments, consisting of curcumin and chitosan in a ratio of 25%, in combination with ZnO and TiO_2_.^32^ Subsequently, it was studied in parallel in both half (12.5%) and double (50%) proportions of 25%. Initially, as one can see, the optimal derivative for the removal of both anionic and cationic dyes is CS/AC@Cur50%. Thus, with an increase in the percentage of curcumin in the CS/AC@ product, the effectiveness increases, proving the beneficial participation of curcumin in the composition of the materials, with the aim being the removal of dyes.

With regard to the optimum solution pH, there is a differentiation between anionic and cationic dyes. In particular, as shown in Figure 1a, for the removal of RB5 (anionic dye) the acidic conditions enhance the removal rates. At pH 2.0 ± 0.1, 93% of RB5 was removed by applying CS/AC@Cur50%, while for CS/AC@Cur12.5% and CS/AC@Cur25%, the removal rates were 74 and 81%, respectively. On the contrary, the degree of removal of MB (cationic dye) increases with pH, with the percentage removal reaching at pH 9.0 ± 0.1 the maximum value, i.e., 47%, 48%, and 54% for CS/AC@Cur12.5%, CS/AC@Cur25%, and CS/AC@Cur50%, respectively. A similar finding was also described recently^18^ with regard to the pH effect. It is obvious that these materials are more efficient for the removal of anionic dyes than cationic dyes and that CS/AC@Cur50% is the optimum in both cases.

**Figure 2:**
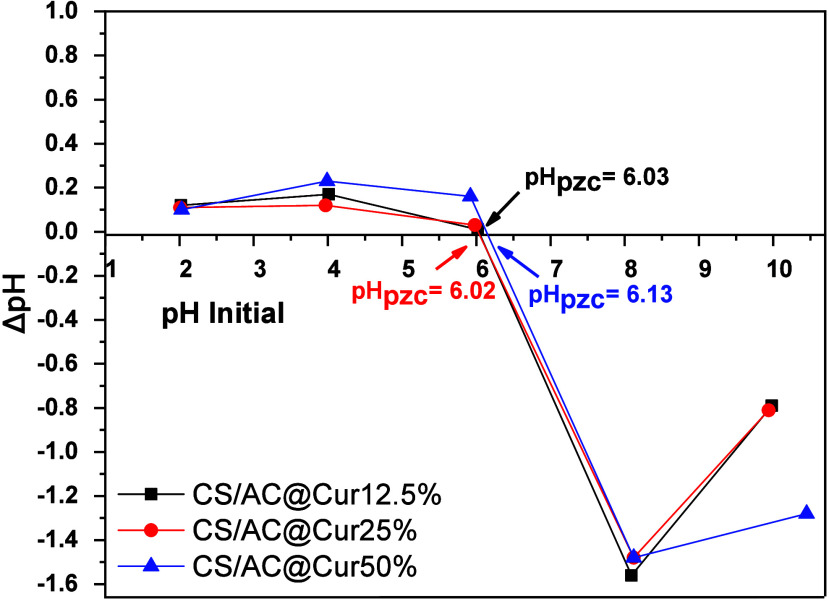
Determination of the pH_pzc_ of CS/AC@Cur adsorbents.

Moreover, in this study, to investigate the surface charge of composite materials, the point of zero charge (pH_pzc_) of each material was measured by applying the pH drift method.^42^ According to the results presented in Figure 2, provided by the ΔpH–pH diagrams, the determined pH_pzc_ values were quite similar, i.e., 6.03, 6.02, and 6.13 for CS/AC@Cur12.5%, CS/AC@Cur25%, and CS/AC@Cur50%, respectively. Therefore, when pH < pH_pzc_, the surface is positively charged, causing the attraction of negatively charged ions, while when pH > pH_pzc_, the surface attracts cations.

**Figure 3:**
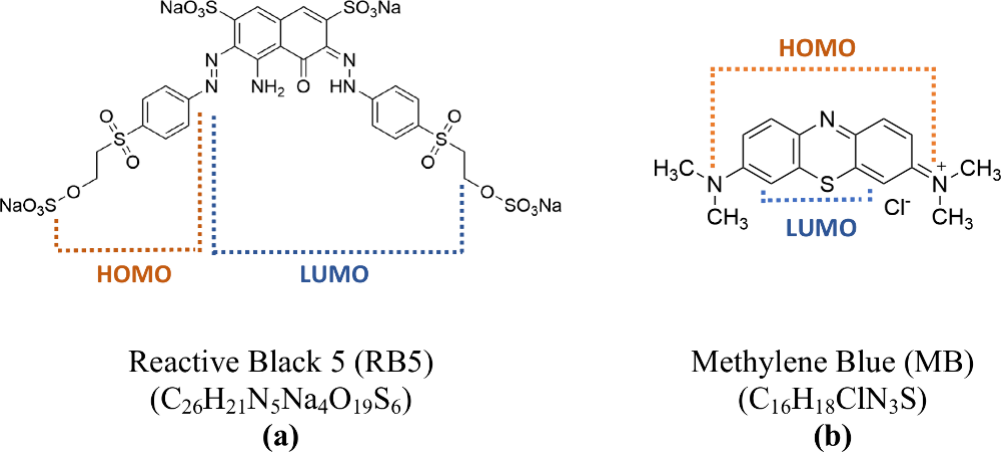
Structures of (a) reactive black 5 (RB5) and (b) methylene blue (MB) dyes (made by the authors using ChemDraw).

As shown in Figure 3a, sulfonate (R-SO_3_^–^) groups are found in the structure of RB5, and reaction R1 takes place in aquatic solutions:
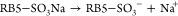
R1

**Figure 4:**
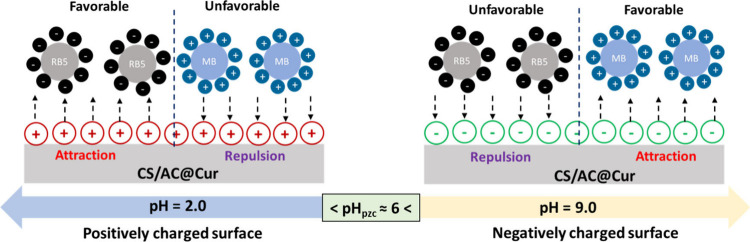
Estimated mechanisms for the removal of anionic RB5 and cationic MB molecules by CS/AC@Cur derivatives.

Therefore, at the optimum pH for RB5 of 2.0 ± 0.1 < pH_pzc_, the surface of CS/AC@Cur derivatives is positively charged and a strong electrostatic attraction with the −SO_3_^–^ groups is expected (Figure 4). However, when pH > pH_pzc_, the negatively charged surface of the adsorbents can lead to electrostatic repulsion between the negative groups of RB5 and CS/AC@Cur, which compete for the adsorption sites, reducing the dye removal rate. Apart from the electrostatic attraction, other interactions may also occur. The positively charged −NH and −OH groups of CS and the −OH units of AC can form hydrogen bonds with the −SO_3_, −SO_2_, −N, =N, and C=O groups of RB5.^43^ Furthermore, due to the presence of AC and Cur on the surface, hydrophobic and π–π interactions may occur between the aromatic rings of the dye molecules and AC and Cur.^44^ This also confirms the reason why the larger quantity of Cur increases the adsorption capacity of RB5.^45^ The highest occupied molecular orbital (HOMO) of RB5 is mainly localized around the C1–C11 benzene ring and partially on the C52–C57 benzene ring; the lowest unoccupied molecular orbital (LUMO) is concentrated on the same C1–C11 benzene ring, as well as on atoms N23 and N24, and the C25–C30 benzene ring, as shown in Figure 3a.^46^ The electron-accepting nature of the LUMO, therefore, can interact with the electron-rich sites on CS/AC@Cur, enhancing the formation of stable complexes. This interaction is quite vital in the effective adsorption of RB5 onto the composite because it stabilizes the dye through π–π stacking and other noncovalent interactions.

Conversely, in Figure 3b, the structure of MB is shown, which is mainly appears in water, in a singly protonated form (MBH^2+^) of the dimethylamino groups.^47^ Consequently, at the optimum pH for MB of 9.0 ± 0.1 > pH_pzc_, the surface of CS/AC@Cur derivatives is negatively charged; therefore, an electrostatic attraction between a positively charged dye and a negatively charged adsorbent is also observed in this case, especially as the pH increases above pH_pzc_. Other possible mechanisms can include n−π (between −OH groups of the adsorbent and aromatic rings of dye molecules), π–π, and hydrophobic interactions (between aromatic rings of Cur and AC and those of MB).^48^ Finally, weaker forces can be present, including Yoshida H-bonding as well as H-bonding.^49^ As seen for RB5, the increased amount of Cur on the adsorbent surface enhances those interactions, resulting in a higher adsorptive efficiency. These interactions are illustrated in Figure 4. Figure 3b shows that the HOMO regions are mainly located on the nitrogen and sulfur atoms, indicating their role as electron donors, while the LUMO regions refer to electron-accepting characteristics.^50^ CS, AC, and Cur, being rich in hydroxyl (−OH) and other functional groups, provide reactive sites for both nucleophilic and electrophilic interactions. The dipole moment and soft nature of MB sustain strong binding on the surface of the composite, especially in a high-pH environment, which is in agreement with adsorption evaluation.^51^

### Effect of the Mass of the Adsorbent

**Figure 5:**
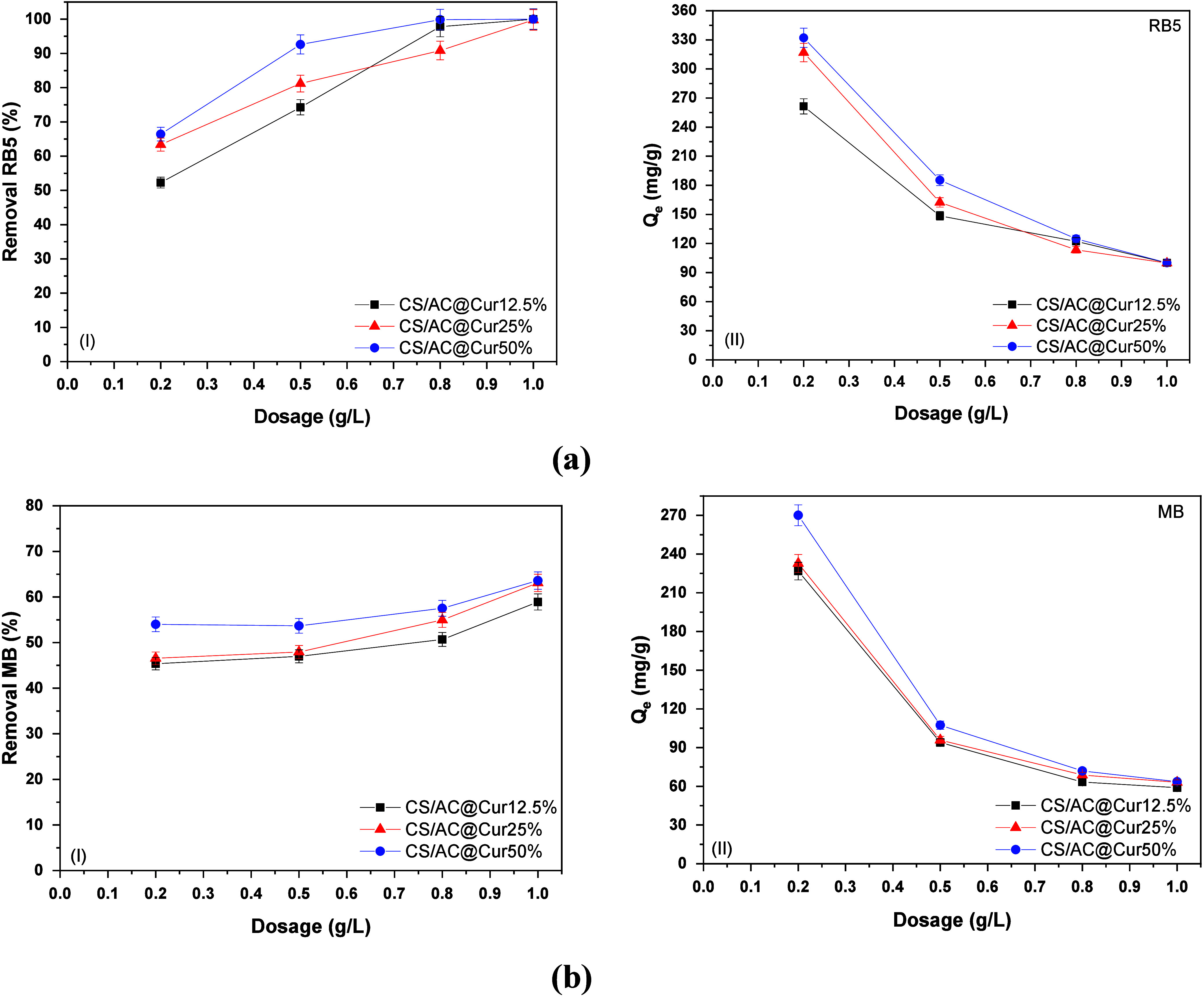
Effect of dosage on (a) RB5 and (b) MB removal, with regard to removal (I) and adsorption capacity *Q*_e_ (II). Experimental conditions: initial concentration of dyes, 100 mg/L; dose of CS/AC@Cur derivatives, 0.2–0.5 g/L; initial solution pH, 2.0 ± 0.1 for RB5 removal and 9.0 ± 0.1 for MB removal; working temperature, 293 K; and duration of the experiment, 24 h.

The effect of the mass of the CS/AC@Cur derivatives on RB5 and MB removal was investigated in the range of 0.2–1.0 g/L, maintaining the initial concentration, dosage, relative pH, and temperature at 100 mg/L, 0.5 g/L, pH 2.0 ± 0.1 for RB5 and pH 9.0 ± 0.1 for MB, and 293 K, respectively, for 24 h, as shown in Figure 5.

The findings indicate an increase in the percentage of removal of both RB5 and MB. Specifically, for RB5 [Figure 5a(I)] the rate increased from 66% to 100% with an increase in the amount of optimum CS/AC@Cur50% from 0.2 to 1.0 g/L. It is worth noting that the removal rate was already high at a lower dose, as with the addition of 0.5 g/L CS/AC@Cur50%, the removal rate was already 93% while the corresponding percentages for CS/AC@Cur12.5% and CS/AC@Cur25% were 52% and 63%, respectively. Similar findings are shown in Figure 5b(I) with regard to the removal of MB, but the overall removal rates remain low, even with the addition of 1.0 g/L CS/AC@Cur50% (64%). This increase can also be found in the recent literature^11^ and can be accredited to the disposal of more adsorption sites on the surface of the adsorbents.

However, as shown in Figure 5a(II) and Figure 5b(II), an increase in the adsorbent dose resulted in a decrease in adsorption capacity, from 332 to 100 mg/g in the case of RB5 [Figure 5a(II)] and from 270 to 64 mg/g in the case of MB, upon addition of the optimum CS/AC@Cur50% adsorbent. This phenomenon can be attributed to the gradient concentration between the solute concentration in the solution and the solute concentration in the surface of the adsorbent, after 24 h, causing a decrease in the equilibrium adsorption capacity. This is because when more mass is added more adsorption sites are added and the amount of adsorbate remains constant, limiting the motivation for additional dye adsorption.^11^

### Effect of Contact Time on the Adsorption Kinetics

**Figure 6:**
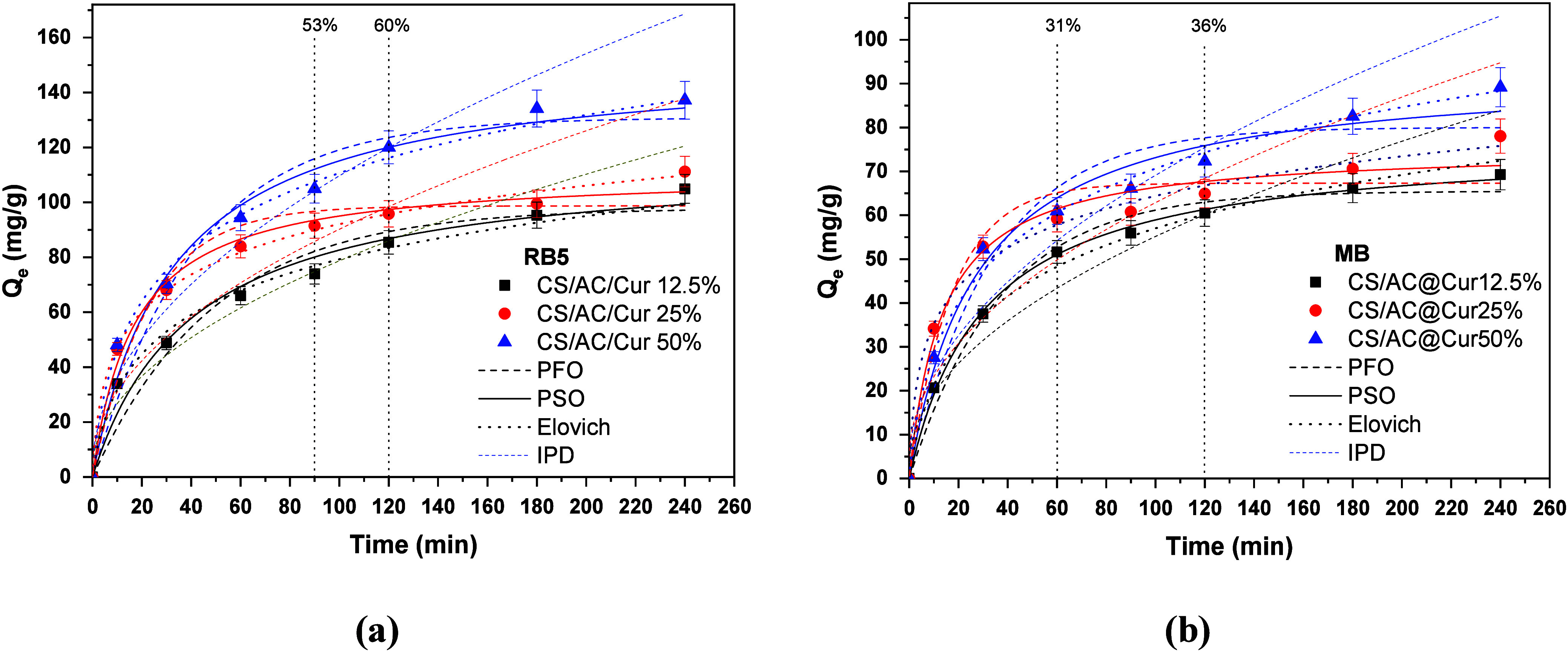
PFO, PSO, Elovich, and IPD kinetic models for (a) RB5 and (b) MB adsorption. Experimental conditions: initial concentration of dyes, 100 mg/L; dose of CS/AC@Cur derivatives, 0.5 g/L; initial solution pH, 2.0 ± 0.1 for RB5 removal and 9.0 ± 0.1 for MB removal; working temperature, 293 K; and duration of the experiments, 0–240 min.

Equilibration time is a factor used to assess, from a practical point of view, how dyes diffuse into the adsorbent. Thus, the effect of contact time on the adsorption of RB5 and MB on CS/AC@Cur derivatives is examined at the optimum pH values of 2.0 ± 0.1 and 9.0 ± 0.1, respectively, by adding 0.5 g/L adsorbents at several time points, such as 0, 10, 30, 60, 90, 120, 180, and 240 min, and the results are shown in Figure 6. It is evident that the rate of removal of dyes increases with experimental time for all of the examined adsorbents. In more detail, the results showed that the rate of removal increased rapidly within 90 min for RB5 (53%) and 60 min for MB (31%) and slowed to 120 min, where 60% of RB5 and 36% of MB were removed with the optimal CS/AC@Cur50% adsorbent. After a contact time of 24 h (1440 min), the phenomenon reaches completion (93% and 54% removal for RB5 and MB, respectively), as shown in Figure 1. This can be explained by the fact that at the beginning there are more active adsorption sites; therefore, electrostatic attraction plays a key role in adsorption. Subsequently, there is a gradual occupation of the adsorption sites, so the adsorption rate decreases.

A crucial factor affecting the adsorption process, as the rate and mechanism of adsorption can be deduced from it, is the study of adsorption kinetics. In this way, the efficiency of adsorption can be evaluated and the adsorption reaction process can be studied.^52^ Experimental data were fitted to commonly applied PFO and PSO nonlinear models as well as Elovich and IPD nonlinear models. The predicted kinetic curves for RB5 and MB dyes on CS/AC@Cur12.5%, CS/AC@Cur25%, and CS/AC@Cur50% are plotted in Figure 6, while the relative calculated kinetic parameters are listed in Table 1.

**Table 1:** Parameters of the PFO, PSO, Elovich, and IPD Models for the Adsorption of RB5 and MB

		PFO			PSO			Elovich			IPD		
material	*Q*_e,exp_ (mg/g)	*K*_1_ (min^–1^)	*Q*_e.cal_ (mg/g)	*K*_1_ (min^–1^)	*Q*_e.cal_ (mg/g)	*R*^2^	*K*_2_ (L mg^–1^ min^–1^)	*b* (g/mg)	*a* (mg g^–1^ s^–1^)	*R*^2^	*K*_IPD_ (mg g^–1^ min^–0.5^)	*C* (mg/g)	*R*^2^
RB5													
CS/AC@Cur12.5%	147.9	0.02	97.9	0.02	97.9	0.951	0.002	0.042	6.78	0.995	10.73	2.28	0.964
CS/AC@Cur25%	162.4	0.04	98.3	0.04	98.3	0.959	0.005	0.032	10.55	0.998	8.66	3.58	0.919
CS/AC@Cur50%	185.2	0.02	107.5	0.02	107.5	0.963	0.002	0.049	18.42	0.999	7.61	2.61	0.970
MB													
CS/AC@Cur12.5%	93.97	0.03	65.49	0.03	65.49	0.971	0.004	0.055	4.02	0.999	6.61	2.99	0.952
CS/AC@Cur25%	95.86	0.06	67.32	0.06	67.32	0.952	0.010	0.076	17.90	0.996	5.80	4.83	0.891
CS/AC@Cur50%	107.36	0.03	80.04	0.03	80.04	0.958	0.004	0.048	6.13	0.996	5.23	2.78	0.953

As one can see, for all prepared adsorbents, the adsorption followed the PSO model and the correlation coefficient (*R*^2^) obtained from each model exhibits a stronger agreement (*R*^2^ > 0.98) with the kinetic data than does the PFO kinetic model. Moreover, the calculated equilibrium adsorption capacities (*Q*_e,cal_) of the PSO kinetic model were closer to the actual equilibrium adsorption capacities (*Q*_e,exp_), providing relatively low Δ*Q* (percent) values; for RB5 removal with the optimal Cs/AC@Cur50%, Δ*Q*_PFO_ = 42.0% and Δ*Q*_PSO_ = 17.6%, and for ΜΒ removal with the same material, Δ*Q*_PFO_ = 25.4% and Δ*Q*_PSO_ = 13.1%. This model^53^ suggests chemisorption as the main mechanism. It is employed to explain adsorption processes that follow second-order kinetics with the premise that the surface of the adsorbent is energetically heterogeneous in nature and hence exhibits evidence of multiple activation energies.

This finding signifies a comprehensive depiction of the adsorption process, suggesting a proportional relationship between the filling intensity of the adsorption centers and the square of the number of vacancies.^54^

### Adsorption Isotherms

To study the adsorption behavior and to regulate the possible process that takes place, adsorption isotherms are applied, as they give relevant information and contribute to the evaluation of the adsorption capacity of the adsorbent. Three most well-known and applied isotherm models were applied to interpret the adsorption behavior of RB5 and MB on the CS/AC@Cur derivatives, namely, Langmuir, Freundlich, and Sips. The latter is used for the mathematically equivalent Langmuir–Freundlich equations, as they offer a precise fit to a given isotherm data set. CS and AC were used as the control samples.

**Figure 7:**
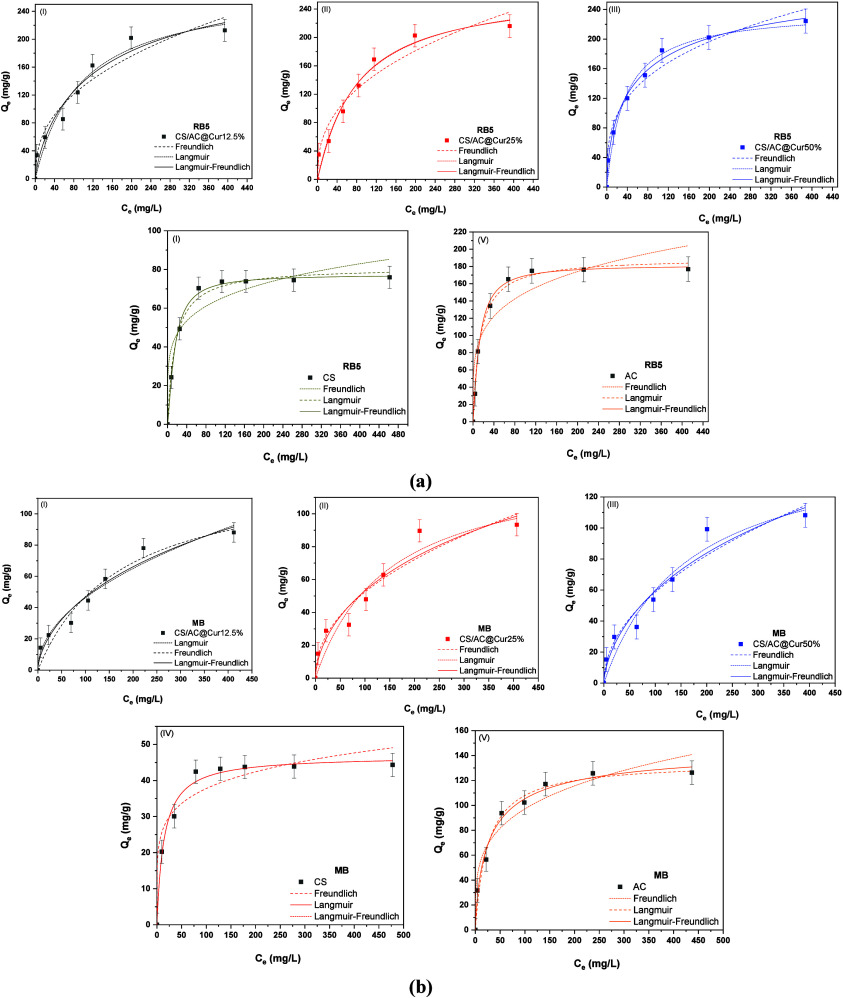
Langmuir, Freundlich, and Sips (Langmuir–Freundlich) isotherm models for (a) RB5 and (b) MB adsorption, with CS/AC@Cur derivatives (I–III) and CS (IV) and AC (V) as control samples. Experimental conditions: initial concentration of dyes, 0–500 mg/L; dose of adsorbents, 0.5 g/L; initial solution pH, 2.0 ± 0.1 for RB5 removal and 9.0 ± 0.1 for MB removal; working temperature, 293 K; and duration of the experiments, 120 min.

Equilibrium adsorption data were obtained by varying the initial dye concentration in the aqueous phase from 0 to 500 mg/L, while the temperature was kept at 293 K and the contact time at 120 min using the optimum for each dye pH. The data are shown in Figure 7; in particular, the equilibrium adsorbed amount [*Q*_e_ (milligrams per gram)] as a function of the concentration of the two dyes at equilibrium [*C*_e_ (milligrams per liter)] and the theoretical curves for the three adsorption isotherm models, using Origin software (OriginPro version 9.0), are plotted. The relative calculated parameters are listed in Table 2. These data were used to explore the relationship between the dye concentration in the aqueous phase and the surface area of the adsorbents.

**Table 2:** Parameters of the Langmuir, Freundlich, and Sips (Langmuir–Freundlich) Isotherm Models

Langmuir Isotherm Model			
material	*Q*_m_ (mg/g)	*K*_L_ (L/mg)	*R*^2^
RB5			
CS/AC+@Cur12.5%	274.15	0.01	0.958
CS/AC@Cur25%	272.35	0.01	0.974
CS/AC@Cur50%	239.15	0.03	0.982
CS	81.17	0.06	0.989
AC	189.83	0.07	0.991
MB			
CS/AC@Cur12.5%	127.75	0.01	0.960
CS/AC@Cur25%	132.21	0.01	0.933
CS/AC@Cur50%	158.37	0.01	0.949
CS	46.81	0.07	0.985
AC	134.74	0.04	0.983

Freundlich Isotherm Model			
material	1/*n*	*K*_F_ (mg/g)·(L/mg)^1/*n*^	*R*^2^
RB5			
CS/AC@Cur12.5%	0.407	20.48	0.954
CS/AC@Cur25%	0.391	22.86	0.953
CS/AC@Cur50%	0.305	39.18	0.978
CS	0.190	26.44	0.890
AC	0.220	54.30	0.867
MB			
CS/AC@Cur12.5%	0.490	4.83	0.968
CS/AC@Cur25%	0.461	6.30	0.942
CS/AC@Cur50%	0.490	6.12	0.955
CS	0.167	17.46	0.928
AC	0.251	30.52	0.944

Langmuir–Freundlich (Sips) Isotherm Model				
material	*Q*_m_ (mg/g)	1/*n*	*K*_LF_ (L/g)	*R*^2^
RB5				
CS/AC@Cur12.5%	328.68	0.776	0.02	0.966
CS/AC@Cur25%	280.74	1.064	0.01	0.974
CS/AC@Cur50%	338.04	0.633	0.07	0.994
CS	77.36	1.333	0.02	0.995
AC	181.74	1.235	0.04	0.995
MB				
CS/AC@Cur12.5%	295.96	0.613	0.01	0.969
CS/AC@Cur25%	264.54	0.599	0.01	0.944
CS/AC@Cur50%	306.28	0.629	0.01	0.957
CS	46.86	0.995	0.07	0.982
AC	148.23	0.763	0.07	0.985

Upon comparison of the data, the adsorption of both RB5 and MB on all CS/AC@Cur derivatives was well fitted by all models, with the Sips (Langmuir–Freundlich) isotherm model showing the highest correlation coefficient (*R*^2^). Therefore, comparatively, the order of the isotherm that best fits the two sets of experimental data in this study is as follows: Sips > Langmuir > Freundlich for RB5 removal and Sips > Freundlich > Langmuir for MB removal. According to the Langmuir–Freundlich isotherm at a low dye concentration, the model would become a Freundlich isotherm model, whereas at a high concentration, it converts into a Langmuir isotherm. It describes the adsorption energy distribution onto the adsorbent’s heterogeneous surface. Moreover, it is known that the Langmuir isotherm is the most suitable for chemisorption, and the Freundlich isotherm is suitable for both physical and chemical adsorption. The combined features are intrinsically present in the Langmuir–Freundlich isotherm, and because of this, it can correlate both physical and chemical adsorption data.

In addition, the calculated isotherm parameter (Table 2) shows that the adsorption capacity (*Q*_m_), according to the Langmuir (two-parameter) isotherm model, is 239 mg/g in the case of RB5 and 158 mg/g for MB with CS/AC@Cur50%. Moreover, according to the Langmuir–Freundlich (three-parameter) isotherm model, the relative *Q*_m_ values are higher, i.e., 338 and 307 mg/g, respectively, probably because there is no complete correlation with the Langmuir isotherm model and therefore the Sips model could be more accurate.^55^

In comparison to the synthesized adsorbent material of this study (CS/AC@Cur), Table 3 presents some revealing materials published in the very recent literature by evaluating parameters that determine adsorption efficiency. Therefore, Table 3 highlights a noteworthy disparity in the adsorption capacities of dyes when using different types of chitosan-based adsorbent and/or curcumin-based composite materials. For instance, cross-linked chitosan-activated charcoal for cationic MB removal^56^ and a chitosan-activated carbon composite for Remazol brilliant blue R (RBBR) removal^57^ exhibited very high adsorption capacities of 625 and 540 mg/g, respectively. However, the adsorbent dose used was quite large in both cases (i.e., 16 and 4 g/L, respectively), potentially increasing the cost of the process. Moreover, several curcumin-based adsorbents, in combination with chitosan (curcumin-conjugated zinc oxide chitosan nanoparticles)^30^ or without chitosan (curcumin-grafted aramid nanofiber-based aerogel films),^26^ are used for dye removal, using smaller doses but indicating also lower adsorption capacities. Therefore, a comparison of the recent literature with the proposed CS/AC@Cur50% of this study reveals that the combination of chitosan, activated carbon, and curcumin in a composite material has never been applied before, and furthermore, its effectiveness has been demonstrated in both cationic (307 mg/g) and anionic (338 mg/g) dyes, adding a dosage of only 0.5 g/L, making it a versatile material.

**Table 3:** Comparison of the Adsorbent Proposed in This Study with Other Adsorbents from the Recent Literature

adsorbent	dye/type	*C*_0_ (mg/L)	dosage (g/L)	pH_init_	*Q*_max_ (mg/g)	removal (%)	ref
AC–CS^a^	Congo red/anionic	10	0.1	6.0	6	70	(58)
chitosan-activated charcoal^b^	MB/cationic	50	16.0	3.0	625	95	(56)
CSWF beads^c^	direct violet-51/anionic	60	10.0	2.0	6	100	(59)
Cs–C^d^	RBBR/anionic	100	4.0	6.0	540	90	(57)
Ch–GA^e^	Congo red/anionic	100	1.5	–	48	–	(60)
Zn(Cur)O–chitosan^f^	Congo red/anionic	11	0.15	–	74	–	(30)
Cur/ANFs^g^	MG/cationic	10	0.2	–	–	94	(26)
CS/AC@Cur50%^h^	RB5/anionic	100	0.5	2.0	338	93	this study
	MB/cationic	100	0.5	9.0	307	54	

a Activated carbon-modified chitosan beads.

b Cross-linked chitosan-activated charcoal.

c Chitosan, sawdust, and magnetic ferrite beads.

d Chitosan-activated carbon composite.

e Chitosan cross-linked with glutaraldehyde aerogel.

f Curcumin-conjugated zinc oxide chitosan nanoparticles.

g Curcumin-grafted aramid nanofiber-based aerogel films.

h Chitosan/activated carbon@curcumin.

### Effect of Temperature on Thermodynamics

**Figure 8:**
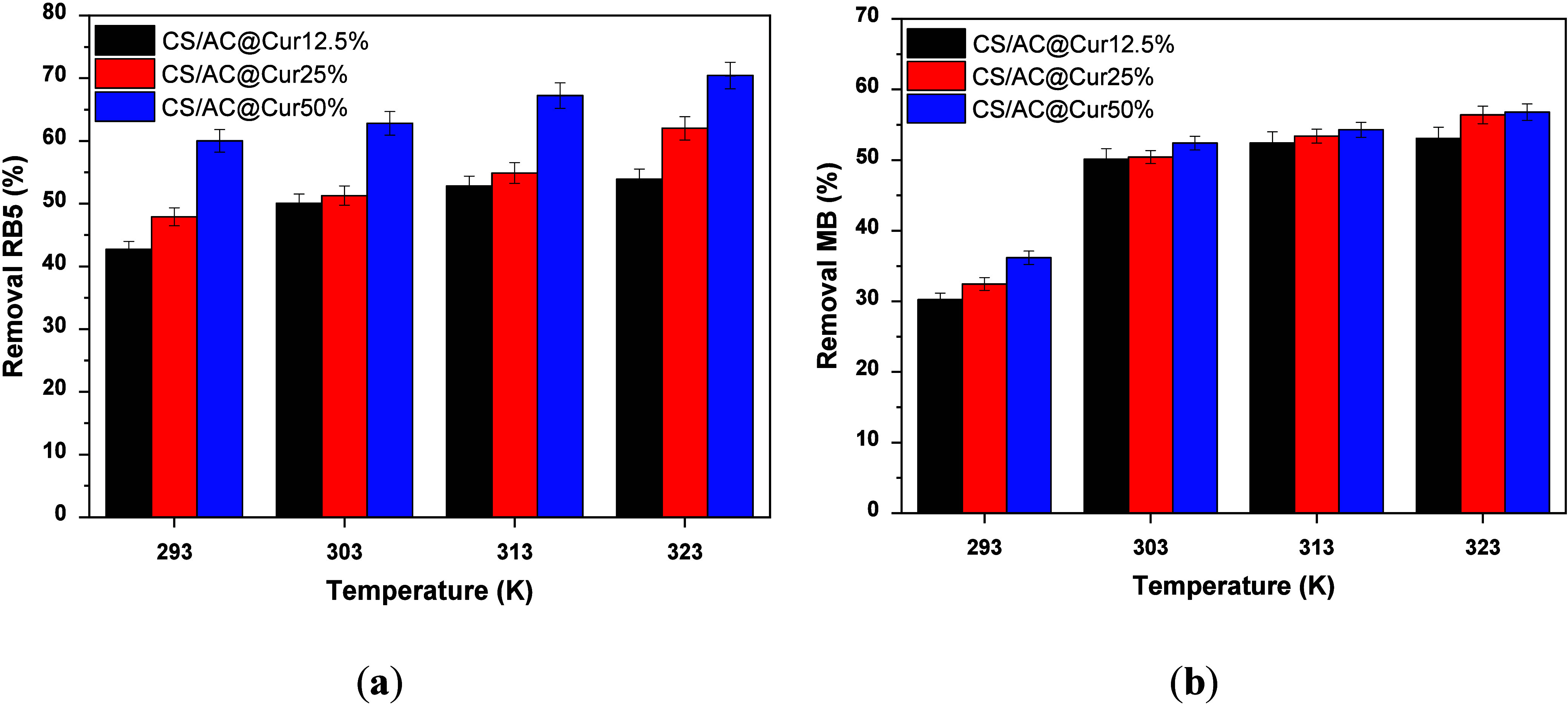
Effect of temperature on (a) RB5 and (b) MB adsorption. Experimental conditions: initial concentration of dyes, 100 mg/L; dose of CS/AC@Cur derivatives, 0.5 g/L; initial solution pH, 2.0 ± 0.1 for RB5 removal and 9.0 ± 0.1 for MB removal; working temperatures, 293, 303, 313, and 323 K; and duration of the experiments, 120 min.

The effect of temperature on the efficiency of CS/AC@Cur derivatives in the elimination of RB5 and MB was considered for 120 min at 293, 303, 313, and 323 K. As shown in Figure 8, the results reveal an increase in removal efficiency with an increase in temperature from 293 to 323 K. For instance, in the case of the optimal CS/AC@Cur50%, the variation was between 60% and 70.4% for RB5 and between 36.2% and 56.8% for MB. This phenomenon can be explained by the fact that increasing the temperature allows more kinetic energy in the adsorbed molecules, thus simplifying access to the internal cavities of the absorbent units.

Thermodynamic studies of the adsorption of RB5 and MB dyes on all CS/AC@Cur derivatives were performed at 303, 313, and 323 K. The main objective of the thermodynamic study is to check the spontaneity of the adsorption process by calculating Δ*G*°. Δ*H*° and Δ*S*° were determined on the basis of the slope and intercept plotting ln(*K*_s_) versus 1/*T*; Δ*G*° was calculated according to eq 9, and all of these values are listed in Table 4. With respect to the results, Δ*G*° is negative for RB5 as well as for MB adsorption, indicating that the adsorption process is spontaneous. Furthermore, this negativity confirms that Δ*G*° is directly proportional to the temperature as it decreases with an increase in temperature. The positive values of Δ*H*° in all cases indicate that the adsorption process is endothermic. Moreover, the values of Δ*S*° are positive for both dyes and can be attributed to the increase in the degree of randomness at the adsorbent–adsorbate interface.

**Table 4:** Thermodynamic Parameters for the Adsorption of RB5 and MB

material	*T* (K)	Δ*G*° (kJ/mol)	Δ*H*° (kJ/mol)	Δ*S*° (kJ mol^–1^ K^–1^)	*R*^2^
RB5					
CS/AC@Cur12.5%	303	–0.030	6.324	0.0210	0.949
	313	–0.240			
	323	–0.449			
CS/AC@Cur25%	303	–0.064	17.775	0.0589	0.955
	313	–0.653			
	323	–1.242			
CS/AC@Cur50%	303	–1.335	14.011	0.0506	0.997
	313	–1.841			
	323	–2.348			
MB					
CS/AC@Cur12.5%	303	–0.038	4.818	0.0160	0.914
	313	–0.198			
	323	–0.359			
CS/AC@Cur25%	303	–0.039	9.766	0.0324	0.999
	313	–0.362			
	323	–0.686			
CS/AC@Cur50%	303	–0.230	7.194	0.0245	0.990
	313	–0.475			
	323	–0.720			

### Effect of Salt Concentration

Real dye wastewater may contain additional contaminants, including various salts such as NaCl, Na_2_CO_3_, NaNO_3_, etc.^61^ These salts may interact with the active sites of the adsorbent and thus hinder the adsorption of the dye to the adsorbent surface. Therefore, it is important to study the effect of the salt concentration on the efficiency of newly synthesized adsorbents. In this study, experiments were conducted with solutions containing sodium chloride (NaCl) and sodium nitrate (NaNO_3_), with varying concentrations of 0.1, 0.3, 0.5, 0.8, and 1 M and a constant dye concentration of 100 mg/L. The experiment was carried out at an optimum pH of 2.0, using 0.5 g/L CS/AC@Cur50% adsorbent, for 3 h at 298 K. The goal was to understand how the concentration of these electrolytes affected the adsorption process.

**Figure 9:**
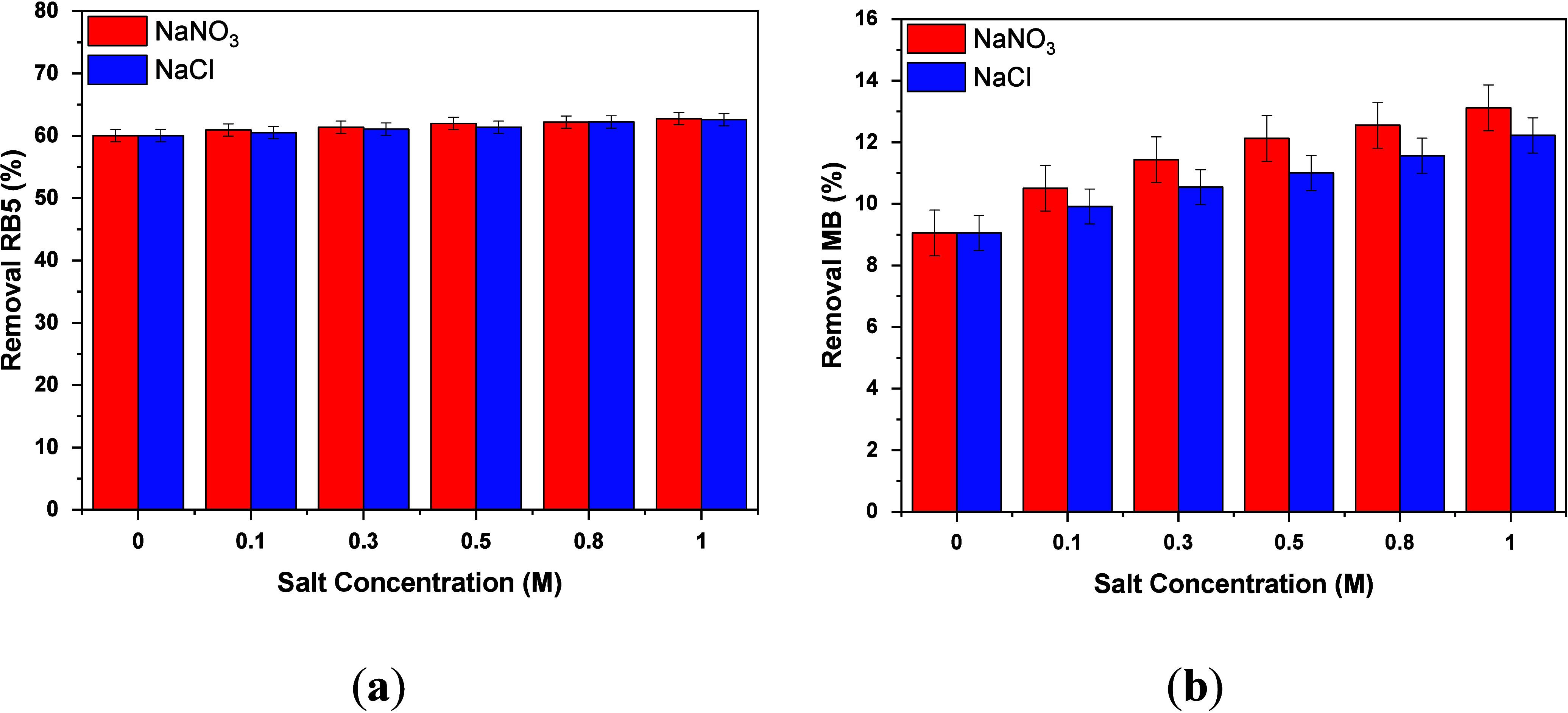
Effect of the concentration of NaCl and NaNO_3_ on (a) RB5 and (b) MB dye removal. Experimental conditions: initial concentration of dyes, 100 mg/L; dose of CS/AC@Cur50%, 0.5 g/L; initial solution pH, 2.0 ± 0 for RB5 removal and 9.0 ± 0.1 for MB removal; salt concentrations, 0.1, 0.3, 0.5, 0.8, and 1 M; working temperature, 293 K; and duration of the experiments, 120 min.

Figure 9 shows the removal rate (percent) for both RB5 (a) and MB (b) dyes at various salt concentrations. As one can see, only a slight increase in the dye removal rate was observed, indicating that the salt concentration does not affect the adsorption of the dye on the CS/AC@Cur50% surface. After 120 min, without the addition of any salt, the rate of removal of RB5 was ∼60%, while after the addition of 1.0 M salt, it increased to only 62.6% when NaCl was added and 62.8% when NaΝO_3_ was added. The obtained results supported the fact that the salt ions coexisting in the RB5 dye solution did not interfere with dye adsorption. Similar findings emerged in the case of MB, while from a 9.1% removal without the addition of salt, at a higher salt concentration (1.0 M), the relevant percentages reached only 12.2% and 13.1% for NaCl and NaΝΟ_3_, respectively.

### Mixtures of Dyeing Solutions

**Figure 10:**
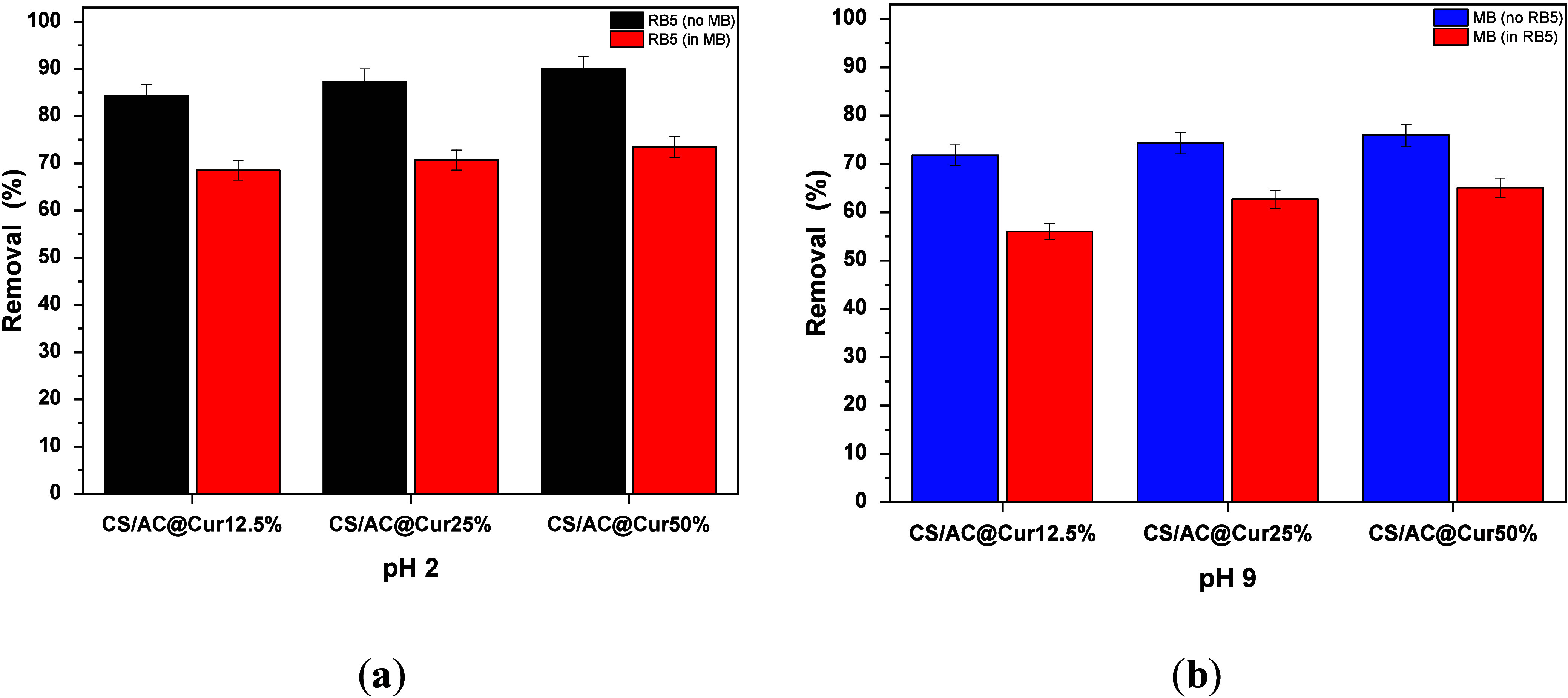
Comparison of CS/AC@Cur adsorbents on (a) RB5 and (b) MB removal in single-component and mixed dye solutions. Experimental conditions: initial concentration of dyes, 20 mg/L; dose of CS/AC@Cur derivatives, 0.5 g/L; initial solution pH, 2.0 ± 0.1 for RB5 removal and 9.0 ± 0.1 for MB removal; working temperature, 293 K; and duration of the experiments, 24 h.

The adsorption of a mixture containing both 20 mg/L basic anionic (RB5) and 20 mg/L cationic (MB) dyes, by CS/AC@Cur derivatives, was investigated, and the relevant results are presented in Figure 10. It should be noted that for the study of RB5 removal, the pH of the solution was adjusted to the optimum value of 2.0 (Figure 10a), and for MB removal, the pH was 9.0 (Figure 10b), applying the same adsorbent dose (0.5 g/L) at 293 K for 24 h. As one can see, with regard to the removal of RB5, with the simultaneous addition of MB to the solution, the rate of removal of RB5, in all cases, is reduced. Specifically, in the case of Cs/AC@Cur50%, from 90% (absence of MB) in the single-dye system, it decreases to 73.5% at pH 2.0. Accordingly, from the results presented in Figure 10b, one can also conclude that the rate of removal of MB decreases slightly from 76% to 65%, with or without RB5, at pH 9.0. Thus, a competitive effect was determined between RB5 and MB, a phenomenon found also in the literature.^62,63^

**Figure 11:**
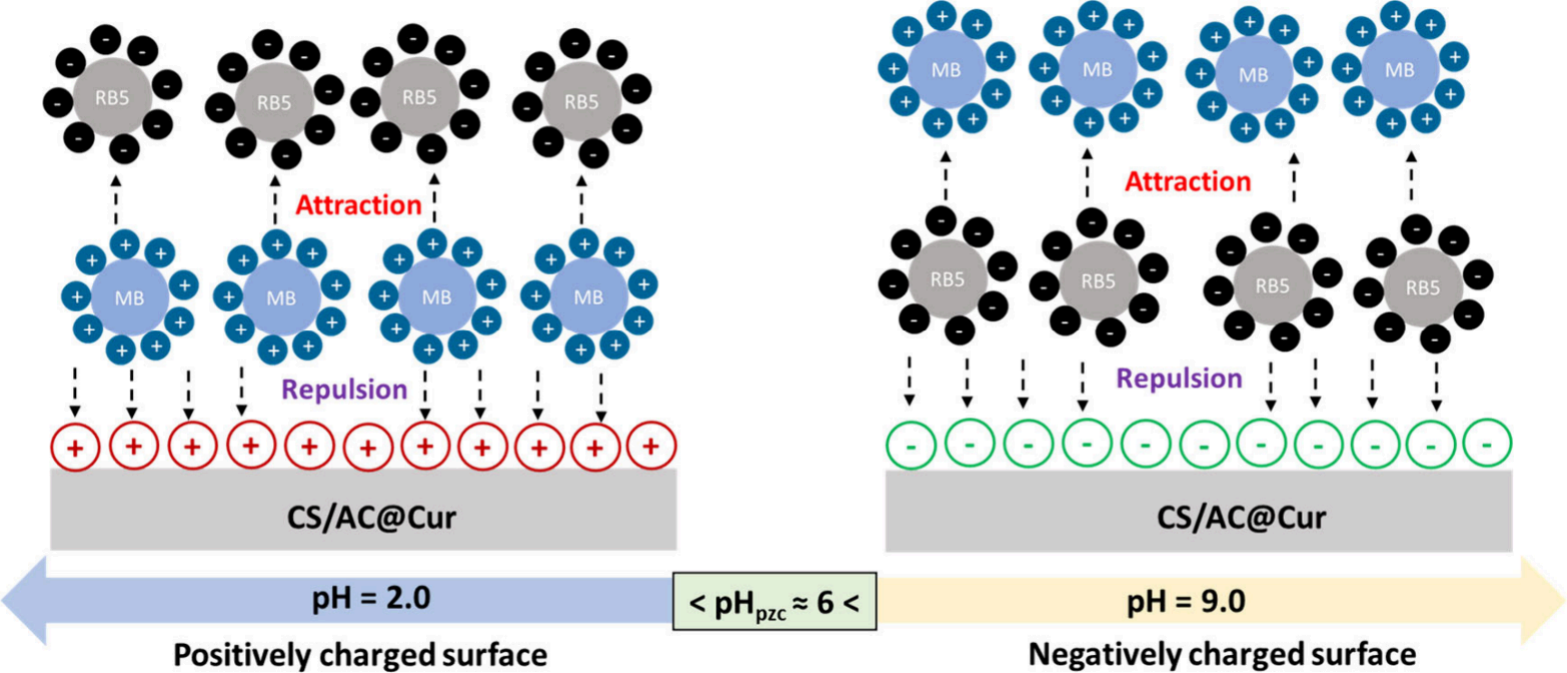
Proposed mechanism for simultaneous removal of anionic RB5 and cationic MB dyes by CS/AC@Cur derivatives in a mixed dye solution.

Moreover, MB, as a cationic dye, exists in its protonated form (MBH^2+^) in water^47^ and RB5, as an anionic dye, comprises the sulfonic groups (Dye-SO_3_^–^).^64^ Therefore, considering a pH_pzc_ of CS/AC@Cur derivatives of ∼6 (Figure 2) and the results presented in Figure 10, we illustrate a proposed mechanism that may occur in these binary systems in Figure 11. Therefore, a competitive effect between the anionic and cationic dyes has to do with electrostatic attractions between the negatively charged SO_3_^–^ groups of RB5 and the positively charged groups of MB, just before adsorption, connected by weak bonds. Then it is repelled by the positively charged surface at pH 2.0 and the negatively charged surface of the material at pH 9.0, thus leading to a reduction in the removal rate. Furthermore, future experiments are needed to examine the effect of other pollutants present in dye wastewater and to perform a corresponding dye adsorption study on real wastewater.

### Regeneration

The regeneration and reuse of adsorbents influence their potential utilization in wastewater treatment. Ten sequential adsorption–desorption cycles were carried out to assess the regeneration and removal efficiencies of the two dyes. CS/AC@Cur50% was chosen as the best-performing adsorbent with CS and AC serving as control samples. The sorption tests revealed that the binding of RB5 and MB required extremely acidic (pH 2.0 ± 0.1) and basic (pH 10.0 ± 0.1) conditions, respectively. As a result, the adsorbed dyes may be retrieved in a reversal environment. Consequently, the eluants for RB5 and MB were aqueous solutions with pH values of 10.0 ± 0.1 and 2.0 ± 0.1, respectively. HCl (0.1 M) and NaOH (0.1 M) solutions were selected to adjust the pH.

**Figure 12:**
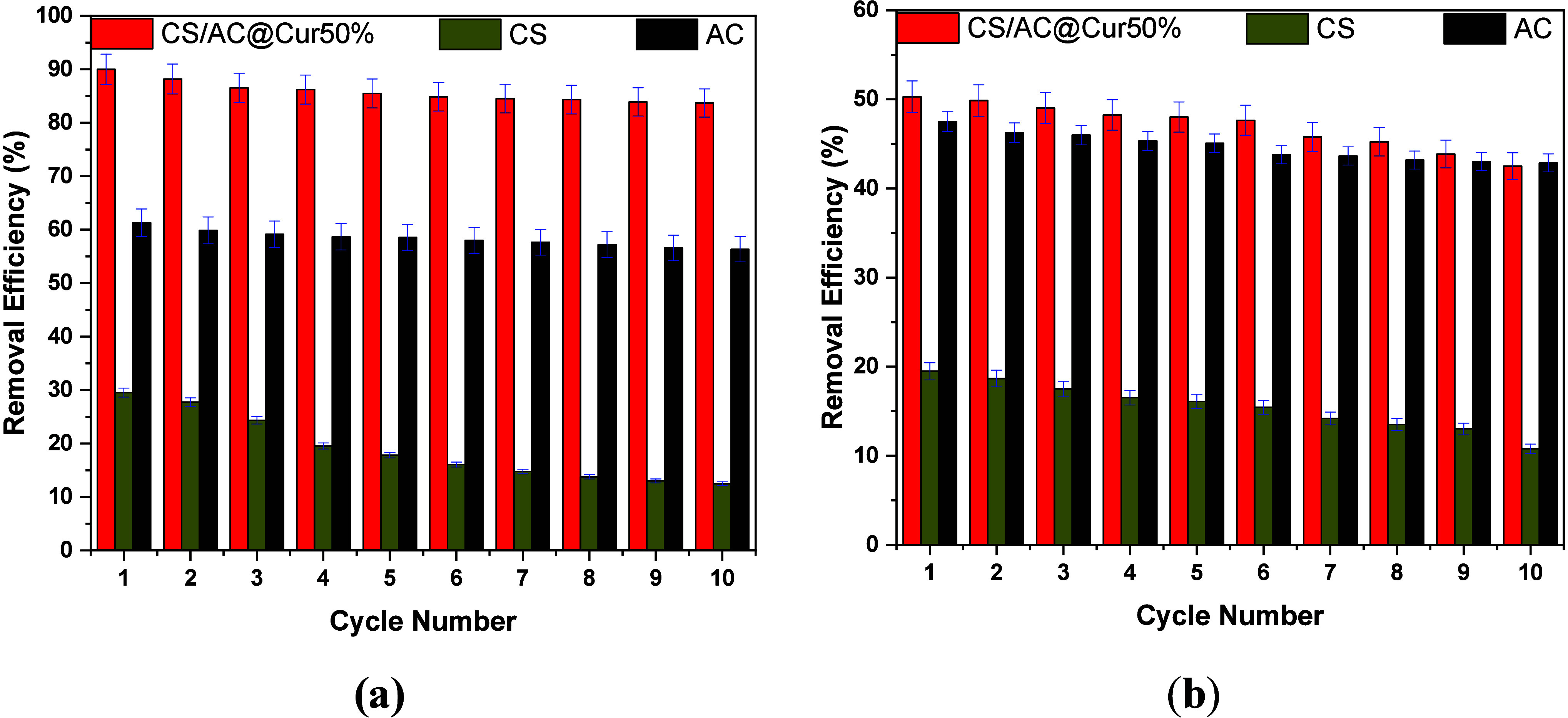
Percentage removal in 10 reuse cycles (adsorption/desorption) for (a) RB5 and (b) MB. Experimental conditions: initial concentration of dyes, 100 mg/L; dose of adsorbents, 0.5 g/L; initial solution pH, 2.0 ± 0.1 for RB5 removal and 9.0 ± 0.1 for MB removal; working temperature, 293 K; and duration of the experiments, 2 h.

Figure 12 exhibits the results of the regeneration experiments. As shown in Figure 12a with regard to RB5, CS/AC@Cur50% maintained its excellent performance, which was slightly reduced after each cycle, losing only ∼7% of its efficiency over the 10 cycles. On the contrary, CS had the largest decrease in removal efficiency, almost 20%, while AC maintained its average performance of ∼58% with the smallest decline.

Figure 12b shows similar results for MB. Again, CS/AC@Cur50% showed the best performance, followed by AC, with a minimal decline of ∼6%. Thus, it is revealed that the incorporation of AC@Cur50% into CS greatly enhances both its adsorption capacity and its mechanical integrity, which is also supported by stability studies. The slight decrease in efficiency can be attributed to the several acid/base washes during the desorption processes, in which a small portion of mass, mainly Cur, was lost.

### Stability

**Figure 13:**
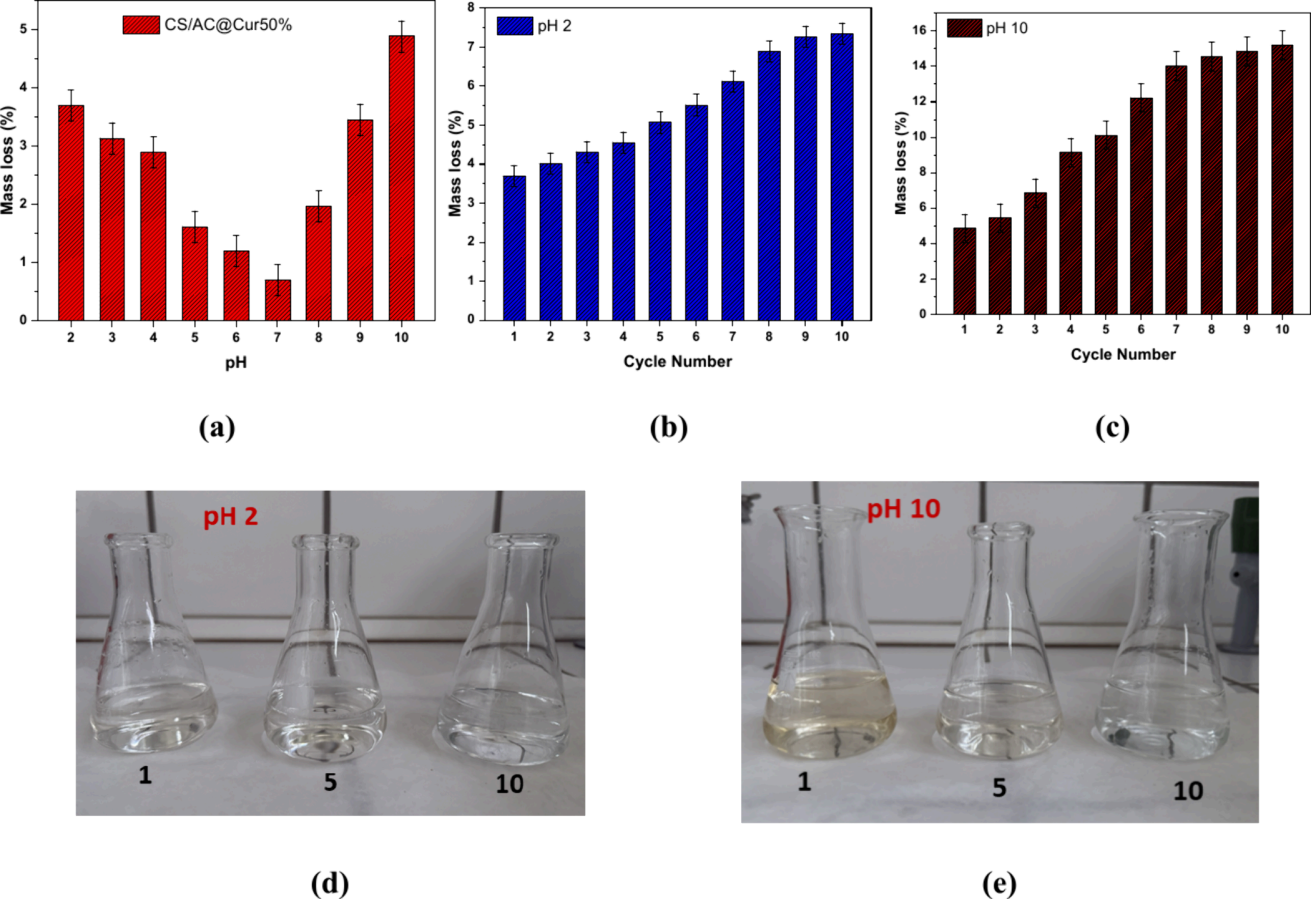
Stability of CS/AC@Cur50% (a) at different pH values, (b) for 10 cycles at pH 2.0 ± 0.1, and (c) for 10 cycles at pH 10. Photographs show that the color of the solutions changes after one (left), five (middle), and 10 (right) cycles for (d) pH 2.0 ± 0.1 and (e) pH 10.0 ± 0.1.

Adsorption and reusability processes rely heavily on pH stability. To examine pH stability, CS/AC@Cur50% was immersed in aqueous solutions with varying pH values (2.0 to 10.0 ± 0.1) for 24 h, and the residual mass was determined after drying. In addition, it was also immersed for 10 consecutive cycles in aqueous solutions of both pH 2.0 ± 0.1 and 10.0 ± 0.1, which were used as eluants for the regeneration experiments. Figure 13a shows that CS/AC@Cur50% is relatively stable at neutral pH (5.0 to 7.0 ± 0.1). At severe pH levels, it maintains its stability, with just 3.7% and 4.9% weight loss at pH 2.0 ± 0.1 and 10.0 ± 0.1, respectively. For this reason, additional experiments were conducted at these pH values. Figure 13b shows that most of the mass loss occurs in the first cycle, 3.7%, and then, it slightly decreases with a maximum of ∼7% in the 10th cycle. On the contrary, in Figure 13c, pH 10.0 ± 0.1 seems to have the greatest impact on the integrity of the adsorbent, losing 10.1% of the total mass in the first five cycles, which was followed by a smaller decrease in the next cycles, stabilizing at 15.2% in the 10th cycle. The latter explains the decrease in the removal efficiency in regeneration experiments. Thus, it is apparent that the pH of the wastewater has no significant effect on the adsorbent’s stability.

As shown in panels d and e of Figure 13, the mass loss is attributed mainly to Cur, which is justified by the color of the solution. In both first cycles, the solution is lightly colored, while in the fifth and 10th cycles, the solutions were clear. Cur can bind to AC through hydrophobic interactions, as proven by FTIR analysis. Also, with the amino units of CS, it can form H-bonds and create Schiff bases that are dynamic and sensitive to pH. However, due to stronger H-bonding and electrostatic interactions in the composite, these Schiff bases can be stabilized. The latter justifies the small loss of Cur in the first cycle, which is then stabilized. Other studies that examined pH stability showed that Cur retention is greatly enhanced when it is combined with chitosan, even in neutral and alkaline media.^65^

### Characterization of Adsorbents

The prepared composites were characterized by N_2_ adsorption measurements to determine the effect of curcumin on the structural properties of the matrix. The domain structural properties (Table 5) such as porosity can be obtained by the type of the produced isotherm following IUPAC categorization. In addition to the shape of the adsorption curve, insights about whether the material is microporous or mesoporous can be determined by analysis of each measurement. The base composite (CS/AC@) presents characteristics of a material containing both micro- and mesopores, while as the percentage of curcumin loading increases, the microporosity disappears. Although it is not obvious by the shape of the plot itself, further analysis by the t-plot method presents linearity passing through the origin, and thus a negligible micropore volume, for the curcumin-loaded composites, while for CS/AC@, there is an intercept providing value by one factor more corresponding to the filling of the <2 nm pores according to the SF (Saito–Foley) model.^66^ However, the portion of the micropores within CS/AC@ seems not to be significant, so the average pore size distribution is on the edge of the microporous/mesoporous range (3.2 nm).

**Table 5:** Physical Properties of the Composite Materials

physical property	CS/AC@	CS/AC@Cur12.5%	CS/AC@Cur25%	CS/AC@Cur50%
BET surface area, SA_BET_ (m^2^/g)	163.1	11.7	12.2	17.8
external surface area, SA_ext_ (m^2^/g)	96.5	11.7	12.2	17.8
DFT pore size (nm)	3.2	6.1	6.2	5.2
cumulative pore volume, *V*_cum_ (cm^3^/g)	0.097	0.027	0.027	0.038
micropore volume, *V*_micro_ (cm^3^/g)	0.0030	0.0025	0.0021	0.0028

**Figure 14:**
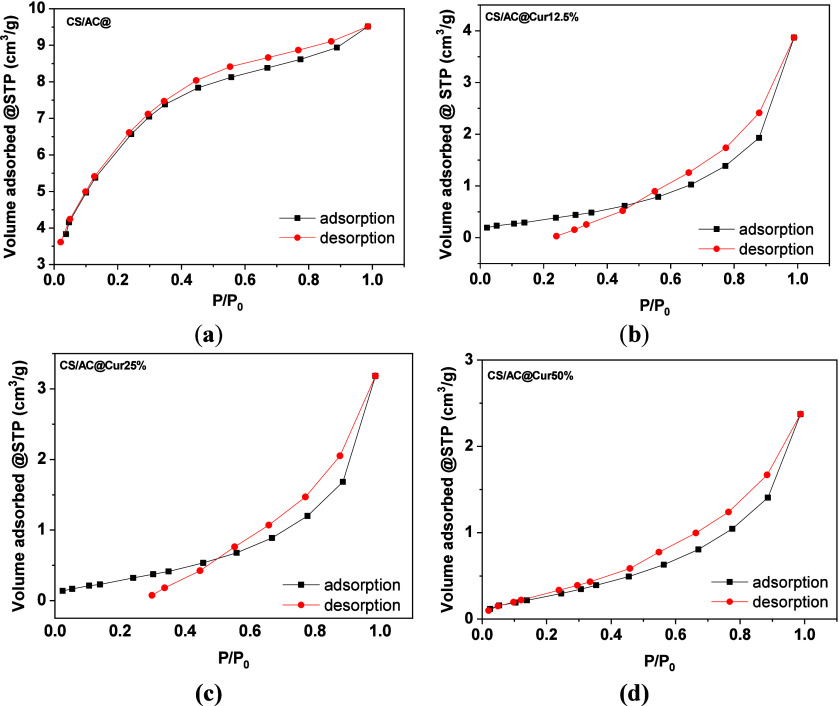
N_2_ adsorption–desorption isotherms of (a) CS/AC@, (b) CS/AC@Cur12.5%, (c) CS/AC@Cur25%, and (d) CS/AC@Cur50% adsorbents.

The lack of microporosity in the curcumin-loaded composites is also supported by the external surface area calculated by the t-method,^67^ where SA_BET_ and SA_ext_ coincide. Specifically, the surface area decreases by an average of 85% when curcumin is present, mostly due to blocking the openings of the AC pores. The isotherm curves of both CS/AC@Cur12.5% and CS/AC@Cur25% (Figure 14b,c) showcase a crossover of the two branches, a phenomenon attributed to expansion and contraction of the material during the adsorption and desorption process.^68^ In the case of 50% curcumin loading (Figure 14d), no crossovers are observed, leading to another insightful finding that there is an optimum percentage of curcumin loading (between 25% and 50%), whereas the structure changes in a way that it becomes more rigid with a slightly larger surface area. Activated carbon’s hydrophobic edges attract the curcumin, resulting in clusters when its percentage is low. While the concentration of curcumin increases, and due to the fact that the most preferable binding sites (AC) are already occupied, curcumin particles are more well distributed along the CS/AC@ matrix, strengthening the composite structure.^69^

**Figure 15:**
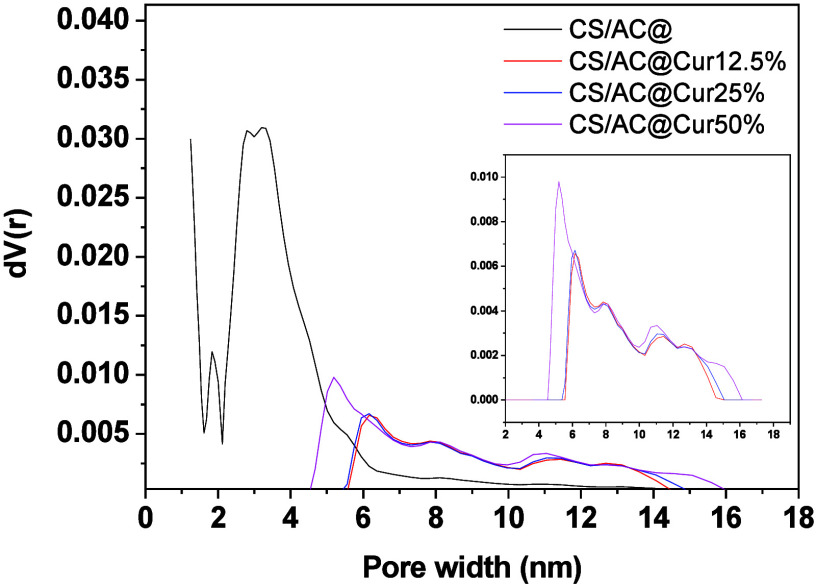
Pore size distribution according to the DFT model. The PSD was derived using QSDFT assuming slit-like pores. The inset shows the PSD of only the curcumin-loaded samples for the sake of clarity and better visibility.

Figure 15 presents the pore size distribution (PSD) of the CS/AC@ composite and the structural alteration as an outcome of the addition of curcumin. As shown in Table 4, the CS/AC average pore size is just ∼3 nm and the PSD graph reveals that it has a distinctive peak just at the end of the micropore region (2 nm), but the following wider peak indicates polydispersity regarding the size of the pores. In fact, the first peak is attributed to AC not fully embedded within the chitosan matrix, while the latter is expected for chitosan.^70^ AC pore blocking due to curcumin can be clearly seen as the PSD curves move to larger sizes, and the sharp peak of the AC disappears. As mentioned above, there is an optimal curcumin percentage above which the composite seems to regain its rigid structure, and this is shown by the shifting back of the main PSD peak for CS/AC@Cur50%.

**Figure 16:**
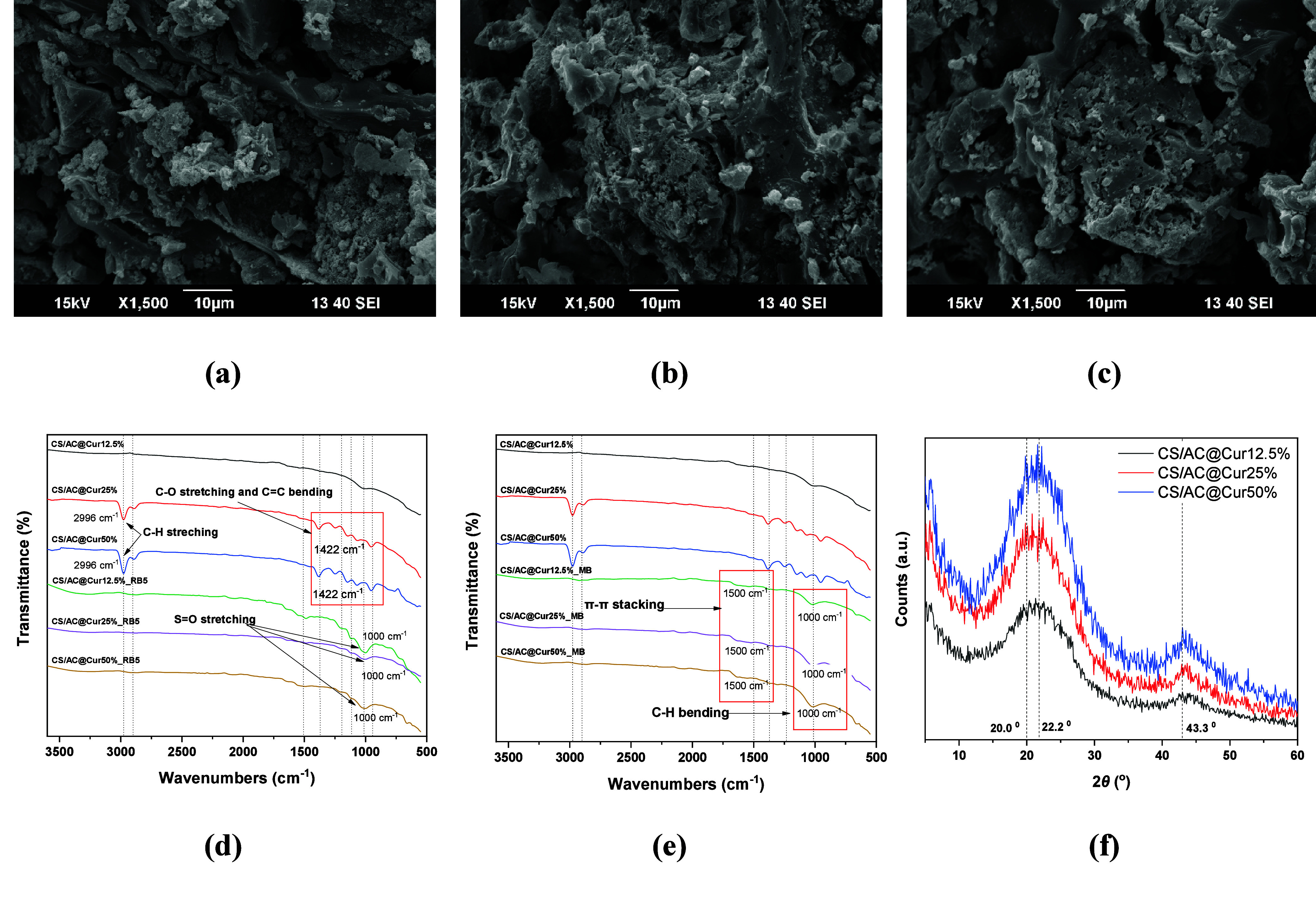
Characterization of the adsorbents. SEM micrographs of (a) CS/AC@Cur12.5%, (b) CS/AC@Cur25%, and (c) CS/AC@Cur50%. FTIR spectra before and after adsorption of (d) RB5 and (e) MB. (f) XRD spectra of CS/AC@Cur12.5%, CS/AC@Cur25%, and CS/AC@Cur50%.

The surface composition alteration of the samples, before and after the adsorption process, was analyzed by FTIR spectroscopy, and the results are illustrated in panels d and e of Figure 16. The spectra of the composites before adsorption support the assumption that curcumin blocks the pores; the peak at ∼3000 cm^–1^ that appears with higher curcumin loading is attributed to the greater number of C–H bonds associated with curcumin’s structure. Curcumin contains multiple aromatic rings and methoxy (−OCH_3_) groups, both of which contribute to C–H stretching vibrations in this region, reflecting the higher concentration of curcumin (25% and 50%) in the composite.^71^ The same is observed in the range of 1000–1400 cm^–1^ attributed to either C–O stretching from the phenolic and methoxy groups or C–O stretching and C=C bending vibrations of the aromatic rings, while in the case of CS/AC@Cur12.5%, the spectrum resembles more that of AC without the pronounced characteristic peaks.

The FTIR spectra after adsorption reveal two different mechanisms of RB5 adsorption. At a low curcumin content (12.5%), the adsorption mechanism based on strong electrostatic interactions is more favored; the −SO_3_ groups of RB5 interact with NH_3_^+^ groups of chitosan leading to S=O stretching (1000–1200 cm^–1^). While the curcumin content increases, the adsorption is favored by the presence of curcumin polar groups, thus enhancing hydrogen bonding with the sulfonate group of RB5, resulting in a reduction in intensity and broadening of the previously pronounced peaks due to restricted movement. In the case of MB, it is more likely to have π–π stacking as the primer mechanism given that MB is a smaller molecule with a planar structure that aligns well with the aromatic rings of curcumin, resulting in the appearance of a broad, low-intensity peak at ∼1500 cm^–1^ as well as some higher-intensity peaks due to out-of-plane C–H bending vibrations of the MB aromatic ring interacting with curcumin’s phenolic groups visible at 1000 cm^–1^.^72^ For both cases, the increased hydrophobicity because of the curcumin concentration enabled preferable conditions, enhancing the adsorption performance. In fact, according to the pH_pzc_ calculation in this study, the higher the curcumin concentration, the more basic the surface of the material becomes (from ∼6.02 for CS/AC@Cur12.5% and CS/AC@Cur25% to ∼6.13 for CS/AC@Cur50%). The latter explains the better performance of the composite for RB5 removal.

The better incorporation of curcumin, at a low percentage, in the CS/AC@ matrix is determined further by SEM micrographs (Figure 16a–c) as well as XRD analysis (Figure 16f). The resulted spectra of the composite at different curcumin concentrations show that as the concentration increases the intensity of the characteristic peaks at ∼20° also does, corresponding to the presence of curcumin and an increase in the crystallinity of the composite. The larger curcumin loading also makes the peaks less broad, even that related to the carbonaceous component of the composite (at ∼43°).^73^ Complementary to the latter, SEM imaging shows a better distribution of curcumin crystals on the matrix (Figure 16a–c). At a low concentration, agglomeration is induced by energetic sites of the CS/AC@ matrix; when the number of the binding sites exceeds the amount of curcumin, there is a tendency to cluster rather than spread.^74^ Regardless of the curcumin concentration, SEM micrographs show a surface with distinct smooth regions and other regions with high porosity, corresponding to chitosan and activated carbon, respectively. As one can see from the presented micrographs, crystal structures within the AC region are more pronounced for a low-curcumin concentration sample (CS/AC@Cur12.5%), while as the percentage increases, the agglomeration is minimized, showcasing a better dispersion of the crystals within the CS/AC@ matrix.

The presence of crystalline structures can be calculated by deploying the Segal method.^75^ The crystalline index (CI, percent) is given by eq 14:
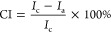
14where *I*_c_ is the intensity of the crystalline phase (the peak with the highest intensity) and *I*_a_ is the intensity of the amorphous phase (the minimum intensity before the crystalline phases).

Except for the crystallinity, another major factor that affects the effective surface area is the crystallite size. The calculation of the crystallite size of each composite was performed with the Scherrer equation (eq 15):^76^
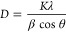
15where *K* is the shape factor (here it is assumed to be 0.9 as it is common for most materials), λ is the wavelength of the X-ray source that in this case is Cu (λ = 1.5405 Å), and β is the full width at half-maximum (fwhm) of the peak in radians. First, 2θ was converted into θ and then into radians.

Table 6 summarizes the results for the composites. From the calculated results, curcumin was incorporated into the CS/AC@ matrix. The increased crystallinity and crystallite size are indicators of the better distribution of curcumin as its percentage increases. In addition, a very subtle shift toward a larger 2θ that is observed can be attributed to lattice space reduction due to hydrogen bonding of curcumin with the matrix.^77^

**Table 6:** Analysis of the XRD Patterns Regarding the Crystallinity Index and Crystallite Size

	CS/AC@Cur12.5%	CS/AC@Cur25%	CS/AC@Cur50%
Parameters			
*I*_a_	321	487	557
*I*_c_	501	769	1016
fwhm (deg)	16.4	15.3	15.2
2θ	21.3	21.1	21.5
Results			
CI (%)	36	37	45
*D* (nm)	4.94	5.28	5.32

## Conclusion

In this study, CS/AC@Cur derivatives were successfully synthesized by combining chitosan, activated carbon, and curcumin, considering the advantages of each additive and their ability as an effective adsorbent for dye elimination. The main findings of this study highlight the adsorption capacity of CS/AC@Cur50%, mostly for anionic (RB5) and less for cationic (MB) dyes, providing 93% removal at pH 2.0 ± 0.1 for RB5 and 54% removal at the optimum pH of 9.0 ± 0.1 due to electrostatic attraction. According to the Sips isotherm model, the maximum adsorption capacities of the CS/AC@Cur50% material were 338 mg/g for RB5 and 307 mg/g for MB. This correlation indicates that the adsorption process is suitable for both physical and chemical adsorption. The investigation of the thermodynamic characteristics confirmed that the adsorption process was endothermic (Δ*H*° > 0) and spontaneous (Δ*G*° < 0). Additionally, the adsorption of RB5 and MB in a mixed solution was investigated, providing a competitive effect between anionic and cationic dyes. Specifically, in the case of Cs/AC@Cur50%, from 90% (without MB) in the single-dye system, it decreases to 73.5% at pH 2.0, in the presence of MB. Furthermore, for all prepared adsorbents, the adsorption followed the Elovich and PSO model with the correlation coefficient (*R*^2^) obtained from each model showing strong agreement (*R*^2^ > 0.98) with the kinetic data. Therefore, the rate-controlling step for all dye adsorption systems may be chemisorption. The results showed that the rate of removal increased rapidly within 90 min for RB5 and 60 min for MB. Also, regeneration experiments showed that CS/AC/@Cur50% can maintain its adsorptive performance for both dyes for ≤10 cycles.

The synthesized materials were fully characterized by FTIR, SEM, BET analysis, and XRD. Specifically, the surface area of CS/AC@ decreased from 163 to 18 m^2^/g for CS/AC@Cur with the addition of curcumin to its structure, mainly due to the blocking of AC pore openings. Furthermore, according to the XRD spectra, as the concentration of curcumin increases, the intensity of the characteristic peaks at ∼20° increases, corresponding to the presence of curcumin and the increase in the crystallinity of the composite. The FTIR spectra of the composites before adsorption support the hypothesis that curcumin blocks the pores and, after the adsorption of the dye, reveal the mechanism of RB5 and MB adsorption.

This research contributes valuable knowledge to the field of water and wastewater treatment, giving a viable and actual solution for controlling dye pollution under environmental conditions. As the proposed material (CS/AC@Cur50%) is applied in binary dye systems, this has potential practical implications for real textile industry wastewater treatment. Future work in this area can rely upon these findings and study optimization policies for CS/AC@Cur derivatives by modifying them to improve their adsorption capacity for cationic dyes. Future experiments are needed both to examine the effect of other pollutants present in dyeing wastewater and to conduct the corresponding dye adsorption study on real wastewater.

## Data Availability

Data will be made available on request.
